# The role of substituted pyridine Schiff bases as ancillary ligands in the optical properties of a new series of *fac*-rhenium(i) tricarbonyl complexes: a theoretical view†

**DOI:** 10.1039/d1ra05737e

**Published:** 2021-11-18

**Authors:** Rosaly Morales-Guevara, Juan A. Fuentes, Dayán Paez-Hernández, Alexander Carreño

**Affiliations:** Universidad Andres Bello, Programa de Doctorado en Físicoquímica Molecular, Facultad de Ciencias Exactas Santiago Chile alexander.carreno@unab.cl d.paez@unab.cl; Laboratory of Organometallic Synthesis, Center of Applied NanoSciences (CANS), Facultad de Ciencias Exactas, Universidad Andres Bello República 330 Santiago Chile alexander.carreno@unab.cl d.paez@unab.cl; Laboratorio de Genética y Patogénesis Bacteriana, Facultad de Ciencias de la Vida, Universidad Andres Bello República 330 Santiago Chile

## Abstract

Over the last few years, luminescent Re(i) tricarbonyl complexes have been increasingly proposed as fluorophores suitable for fluorescence microscopy to visualize biological structures and cells. In this sense, incorporating an asymmetrical pyridine Schiff base (PSB) as the ancillary ligand strongly modifies the staining and luminescent properties of Re(i) tricarbonyl complexes. In this work, we analyzed two series of Re(i) tricarbonyl complexes with their respective PSB ligands: (1) *fac*-[Re(CO)_3_(2,2′-bpy)(PSB)]^1+^ and (2) *fac*-[Re(CO)_3_(4,4′-bis(ethoxycarbonyl)-2,2′-bpy)(PSB)]^1+^, where the PSB exhibits substitutions at positions 4 or 6 in the phenolic ring with methyl or halogen substituents. Thus, we performed computational relativistic DFT and TDDFT studies to determine their optical properties. The ten complexes analyzed showed absorption in the visible light range. Furthermore, our analyses, including zero-field splitting (ZFS), allowed us to determine that the low-lying excited state locates below the ^3^LLCT states. Interestingly, seven of the ten analyzed complexes, whose corresponding PSB harbors an intramolecular hydrogen bond (IHB), exhibited luminescent emission that could be suitable for biological purposes: large Stokes shift, emission in the range 600–700 nm and *τ* in the order of 10^−2^ to 10^−3^ s. Conversely, the three complexes lacking the IHB due to two halogen substituents in the corresponding PSB showed a predicted emission with the lowest triplet excited state energy entering the NIR region. The main differences in the complexes' photophysical behavior have been explained by the energy gap law and time-resolved luminescence. These results emphasize the importance of choosing suitable substituents at the 4 and 6 positions in the phenolic ring of the PSB, which determine the presence of the IHB since they modulate the luminescence properties of the Re(i) core. Therefore, this study could predict Re(i) tricarbonyl complexes' properties, considering the desired emission features for biological and other applications.

## Abstract 2

Over the last few years, luminescent Re(i) tricarbonyl complexes have been increasingly proposed as fluorophores suitable for fluorescence microscopy to visualize biological structures and cells.

## Graphical abstract

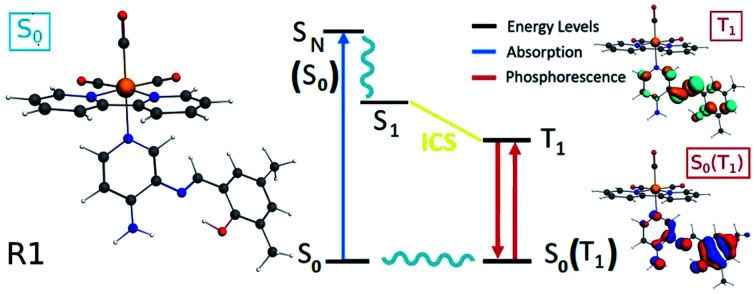

## 1. Introduction

Complexes based on a Re(i) tricarbonyl core with a dinitrogenated ligand (*N*,*N* such as 1,10-phenanthroline or 2,2′-bipyridine derivatives) in the equatorial position, and halogen or pyridine derivatives as ancillary ligands (X), have been extensively used for diverse applications, including solar cells,^1,2^ catalysis,^3–8^ and more recently, biological applications,^9–17^ including staining of bacteria, yeasts (walled cells), and proteins separated by SDS-PAGE.^18,19^

Photophysical attributes of *fac*-[Re(i)(CO)_3_(*N*,*N*)X]^*n*^ complexes (where *n* is 0 or 1+) can be modulated by combining the (*N*,*N*) and X ligands.^10,17,19^ A particular combination of ligands can affect the metalcore due to their donor or acceptor properties.^20–24^ Interestingly, ligands choice affects the photophysical properties and impacts the biological effect, such as therapeutical applications or antimicrobial properties, including protein stain.^18,25,26^

Over the last few years, luminescent Re(i) tricarbonyl complexes have been increasingly proposed as fluorophores suitable for fluorescence microscopy to visualize biological structures and cells.^15,27–29^ Their chemical stability,^30^ enhanced photostabilities (leading to lower photobleaching),^31^ and a relatively good cellular uptake^15,29,32,33^ represent attractive features in these complexes. The use of (*N*,*N*) ligands such as 2,2′-bipyridine (2,2′-bpy) or derivatives can modulate photophysical and biological properties of Re(i) complexes. For instance, when to Re(i) complexes harboring the same ancillary ligand (Br: bromide) where compared, it has been shown that *fac*-Re(i)(CO)_3_(2,2′-bpy)(Br) presents a maximum absorption at 383 nm,^34^ whereas *fac*-Re(i)(CO)_3_(4,4′-bis(ethoxycarbonyl)-2,2′-bpy)(Br) exhibits maximum absorption at 419 nm.^35,36^ In addition, it has been stated that *fac*-Re(i)(CO)_3_(2,2′-bpy)(Br) can stain yeasts (eukaryotic walled cells), whereas *fac*-Re(i)(CO)_3_(4,4′-bis(ethoxycarbonyl)-2,2′-bpy)(Br) is unable to stain the same cells under similar experimental condition.^19,37^ All this evidence shows that relatively small substitution can affect both photophysical and staining properties.^19^

The use of dinitrogenated ligands (*N*,*N*) allows incorporating ancillary ligands (X), which also modulate the complex properties. In this sense, the addition of an asymmetrical pyridine Schiff base (PSB) as the ancillary ligand strongly modifies the photophysical properties, but also the staining properties of Re(i) tricarbonyl complexes.^19,37^ Schiff bases are aldehyde- or ketone-like compounds, where the carbonyl group is replaced by an azomethine (–C=N–) group.^38^PSB are constituted by two rings (a pyridine and a phenolic ring) connected by the azomethine group. In particular, some PSBs present intramolecular hydrogen bond (IHB) that provides stability.^39^ This kind of PSBs (in particular, (*E*)-2-((3-amino-pyridin-4-ylimino)-methyl)-4,6-di-*tert*-butylphenol) has been used as ancillary ligands of Re(i) tricarbonyl complexes, showing promising results as fluorophores for fluorescent microscopy, especially with walled cells (yeasts and bacteria).^35,37^ Thus, as stated above, the *fac*-Re(i)(CO)_3_(4,4′-bis(ethoxycarbonyl)-2,2′-bpy)(Br) is unable to stain yeasts; nevertheless, the similar complex *fac*-Re(i)(CO)_3_(4,4′-bis(ethoxycarbonyl)-2,2′-bpy)((*E*)-2-((3-amino-pyridin-4-ylimino)-methyl)-4,6-di-*tert*-butylphenol)^1+^, where the Br was substituted by (*E*)-2-((3-amino-pyridin-4-ylimino)-methyl)-4,6-di-*tert*-butylphenol, showed a differential staining ability, remaining retained in the cell nucleus.^37^

**Scheme 1:**
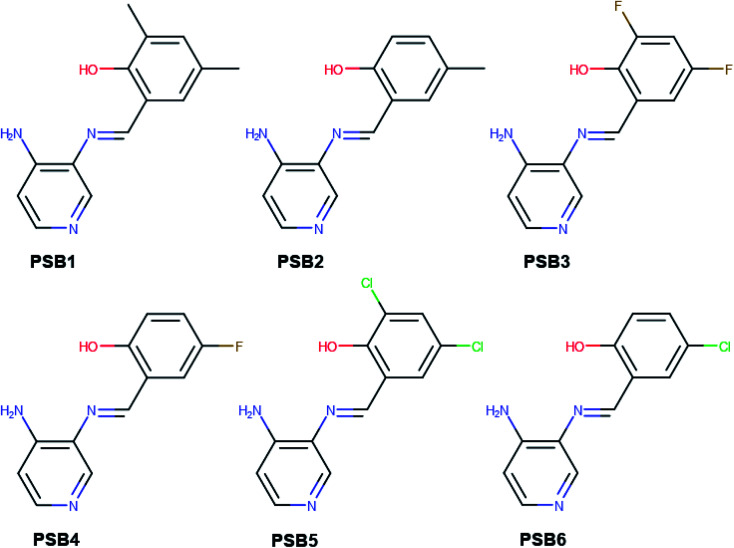
Chemical structure of the pyridine Schiff bases: (*E*)-2-(((4-aminopyridin-3-yl)imino)methyl)-4,6-dimethylphenol (PSB1), (*E*)-2-(((4-aminopyridin-3-yl)imino)methyl)-4-methylphenol (PSB2), (*E*)-2-(((4-aminopyridin-3-yl)imino)methyl)-4,6-difluorophenol (PSB3), (*E*)-2-(((4-aminopyridin-3-yl)imino)methyl)-4-fluorophenol (PSB4), (*E*)-2-(((4-aminopyridin-3-yl)imino)methyl)-4,6-dichlorophenol (PSB5), and (*E*)-2-(((4-aminopyridin-3-yl)imino)methyl)-4-chlorophenol (PSB6).

**Fig. 1:**
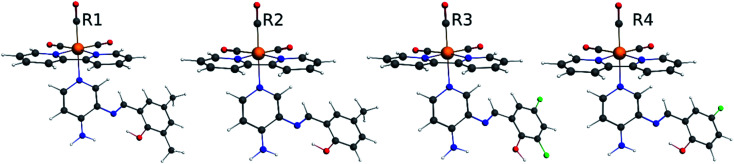
Optimized geometry of the Re(i) tricarbonyl complexes R1 to R4 (series 1).

**Fig. 2:**
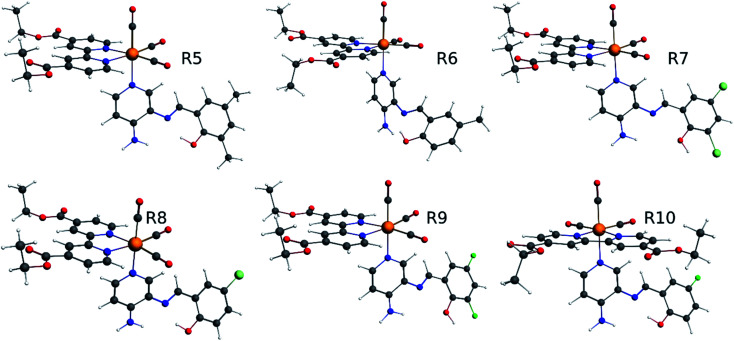
Optimized geometry of the Re(i) tricarbonyl complexes R5 to R10 (series 2).

Our group has already synthesized and characterized some PSBs, derivated from (*E*)-2-((3-amino-pyridin-4-ylimino)-methyl)-4,6-di-*tert*-butylphenol,^35^ such as (*E*)-2-(((4-aminopyridin-3-yl)imino)methyl)-4,6-difluorophenol (PSB3), (*E*)-2-(((4-aminopyridin-3-yl)imino)methyl)-4-fluorophenol (PSB4), (*E*)-2-(((4-aminopyridin-3-yl)imino)methyl)-4,6-dichlorophenol (PSB5), and (*E*)-2-(((4-aminopyridin-3-yl)imino)methyl)-4-chlorophenol (PSB6) (Scheme 1). We previously characterized these PSBs regarding their structural, optical, electronical and antimicrobial properties.^39–42^ On the other hand, the PSBs (*E*)-2-(((4-aminopyridin-3-yl)imino)methyl)-4,6-di-methylphenol (PSB1), and (*E*)-2-(((4-aminopyridin-3-yl)imino)methyl)-4-methylphenol (PSB2), to our knowledge, are new (Scheme 1). In the present study, we worked with two series of Re(i) tricarbonyl complexes with their respective PSB ligands (PSB1 to PSB6, Scheme 1), which are summarized in Table S1:† (1) *fac*-[Re(CO)_3_(2,2′-bpy)(PSB)]^1+^ (Fig. 1, four complexes) and (2) *fac*-[Re(CO)_3_(4,4′-bis(ethoxycarbonyl)-2,2′-bpy)(PSB)]^1+^ (Fig. 2, six complexes). We determined the optical properties of all complexes by computational relativistic DFT and TDDFT analyses,^43–47^ showing that the ten complexes presented absorption in the visible light range. To perform these calculations, we used the B3LYP exchange–correlation functional^48–50^ with the triple-ζ quality double plus polarization function (TZ2P)^51^ to include the polarized effects for all atoms.^35^ We found that the complexes harboring PSB that could form IHB (*i.e.*PSB1, PSB2, PSB4, and PSB6) exhibited luminescent emission suitable for biological purposes: large Stokes shift, emission in the range 600–700 nm and *τ* in the order of 10^−2^ to 10^−3^ s. By contrast, complexes constituted by PSB lacking the IHB (*i.e.*, PSB3 and PSB5) exhibited a predicted emission with the lowest triplet excited state energy entering the NIR region. Since positions 4 and 6 in the phenolic ring in the PSB participate in forming the IHB, we postulate that choosing suitable substituents at these positions could be relevant to modulate the photophysical behavior of this kind of complexes.

## 2. Computational methods

All the proposed structures were optimized in the framework of the density functional theory (DFT) using the Amsterdam Density Functional (ADF) code,^52^ where the scalar and the spin–orbit relativistic effects were incorporated using the two-component Hamiltonian with the zeroth-order regular approximation (ZORA).^53–55^ For relativistic calculations performed on organometallic chemical systems including heavy atoms (such as rhenium), ZORA expands the Dirac equation in *E*/(2*mc*^2^ − *V*), and contains corrections in 1/*c*^2^ to all orders, and can be written as the sum of a scalar relativistic (SR) part and a SOC part.^56^

The quality of the obtained minimum was corroborated with frequency calculations, being positive in all proposed complexes. The hybrid B3LYP exchange–correlation functional, using the local density approximation to the correlation functional (which stands for Becke, 3-parameter, Lee–Yang–Parr),^48–50^ was used with the standard Slater type orbital (STO) basis set with the triple-ζ quality double plus polarization function (TZ2P)^51^ to include the polarized effects for all atoms. Implicit solvation effects on the geometry and optical properties were considered using a dielectric continuum model (COSMO)^57–60^ with dichloromethane as solvent to compare our results with previously reported experimental data with similar Re(i) complexes.^34–36,61^

**Fig. 3:**
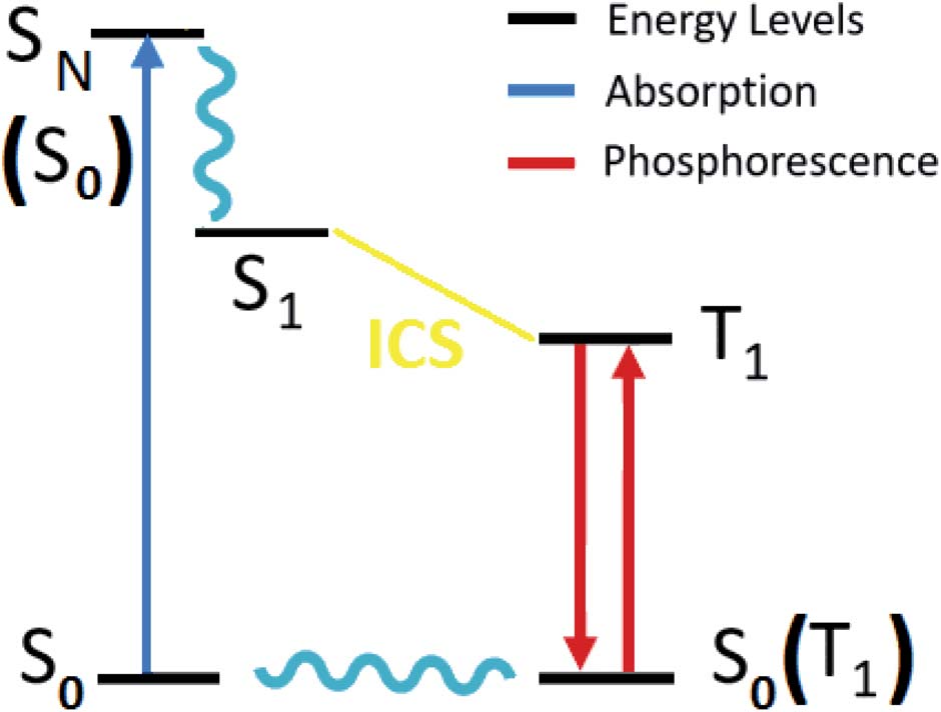
Diagram of the electronic states involved in the theoretical protocol used to predict optical properties of Re(i) complexes *via* TDDFT.

Excitation energy and absorption spectra were calculated using the scalar relativistic time-dependent density functional theory (SR-TDDFT), considering 60 excitations with ground state optimized and the vertical transition to excited singlet state (Fig. 3).^62,63^ A similar calculation considering 40 excitations (with triplet state optimized) was used as the basis for the self-consistent two-component spin–orbit coupling TDDFT (SOC-TDDFT)^64^ within the ZORA Hamiltonian, where 6 spin-mixed excitations were calculated using the SR-TDDFT results as input.^27,65,66^ Since the T_1_ state is the lowest in the triplet manifold, the T_1_ geometry can be optimized like a regular ground state. However, with triplet spin multiplicity (unrestricted DFT) in a regular geometry optimization *via* the most popular generalized gradient approximated potentials GGA with nonlocal exchange and correlation corrections with the PBE functional and the SR-ZORA Hamiltonian without symmetry to reproduce the possible symmetry breaking in the excited triplet state. The emission analysis of R1 to R10 was studied by the model of spin-forbidden phosphorescence (vertical transition from triplet state optimized to singlet ground state T_1_ → S_0_) (Fig. 3).^62,63^ Usually, the triplet state is lower in symmetry than the ground state; thus, it is not recommended to consider symmetry restrictions. In this part of the work, we are interested in electronic transitions between the visible and ultraviolet regions.

The radiative rate *k*_*i*_ and radiative lifetime *τ*_*i*_ from sub-state *i* (*i* = 1, 2, 3) of the T_1_ state to the ground state were calculated from the excitation energy Δ*E*_*i*_ and the transition dipole moment *M*^*i*^ with SOC included (eqn (1)).^67,68^1
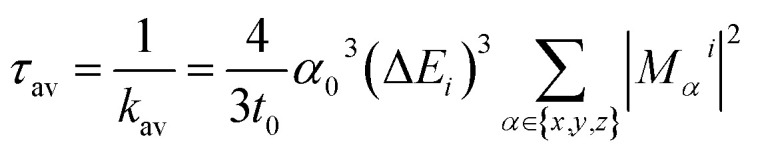
where *t*_0_ = (4π*ε*_0_)^2^ℏ^3^/*m*_e_*e*^4^ and *α*_0_ the Sommerfeld's constant (7.297 × 10^−3^, the fine structure constant, that quantifies the strength of the electromagnetic interaction between elementary charged particles),^69,70^*i* are the triplet sub-states (*i* = 1, 2, 3), Δ*E*_*i*_ is the excitation energy, and *M*^*i*^ is the transition dipole moment.^71^ In a dichloromethane medium, these are corrected for the refractive index *n* according to the Strickler–Berg relationship.^72^*τ*_*i*_ is then an average over the three sub-states as follow in eqn (2):2
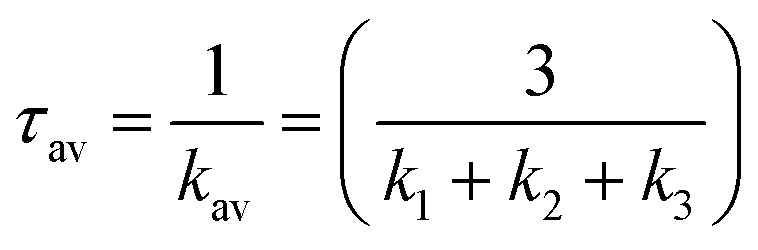


From the Boltzmann distribution, the *τ*_av_ can be obtained as follows in eqn (3):^73–75^3
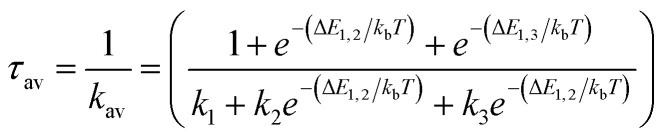
*k*_b_ is the Boltzmann constant, and *T* is the temperature (300 K) used for the calculated lifetimes.

All calculated radiative rate *k*_*i*_ (or radiative lifetime *τ*_*i*_) was multiplied (or divided) by the square of the refractive index of dichloromethane.

### 2.1 Zero-field splitting

**Fig. 4:**
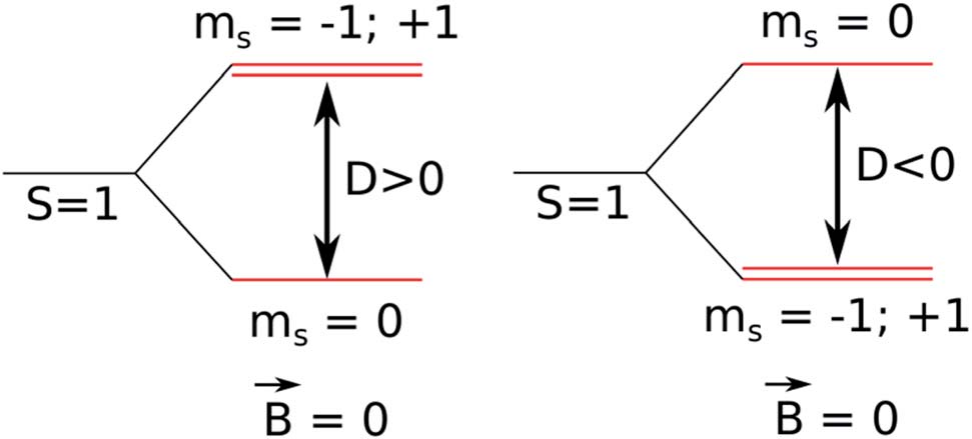
Schematic diagram of a triplet state zero-field splitting (ZFS).

The luminescence properties of organometallic Re(i) tricarbonyl complexes are crucially affected by relativistic effects.^47,76^ In a non- or scalar-relativistic picture, triplet states are three-fold degenerate.^43,77^ The spin–orbit effect on this degeneracy can be quantified by the zero-field splitting (ZFS) resulting from the dipolar interaction between magnetic moments of unpaired electrons (Fig. 4), which is relevant to clarify the nature of the emissive state.^78,79^

ZFS, in combination with emission lifetime (*τ*_r_), is an important parameter to characterize the first triplet excited state of these transition metal complexes.^80,81^

## 3. Results and discussion

### 3.1 Geometry structures in the ground state

The optimized structures of all the Re(i) complexes are shown in Fig. 1 (series 1) and Fig. 2 (series 2). These structures and the geometry parameters were obtained by comparing the experimental data of similar Re(i) complexes, including *fac*-[Re(CO)_3_(4,4′-dimethyl-2,2′-bpy)(*E*)-2-(((4-aminopyridin-3-yl)imino)methyl)-4,6-di-*tert*-butylphenol)]^1+^,^82^ and *fac*-Re(CO)_3_(4,4′-bis(ethoxycarbonyl)-2,2′-bpy)Br.^35,82^ As depicted in Fig. 1 and 2, the Re(i) adopts a distorted octahedral coordination geometry with the three carbonyls of all complexes distributed with facial coordination. The equatorial Re–C (around 1.932 Å) and axial Re–C (around 1.939 Å) bond distances showed no significant differences. Experimental results obtained for similar Re(i) complexes show equivalent distances.^11,17,83^ In the case of C=O carbonyl ligands, the axial C=O distance (around 1.159 Å) is shorter than that of the equatorial (around 1.162 Å) bond (Tables S2 and S3 in the ESI†). This result is attributed to the ligand-to-metal back bonding effect in both axial and carbonyl ligands at equatorial positions.^84–86^ The Re(i)–(*N*,*N*) ligand (where *N*,*N* is 2,2′-bpy or 4,4′-bis(ethoxycarbonyl)-2,2′-bpy) distances are approximately 2.192 Å. These distances are shorter than the Re(i)–PSB (ancillary ligand) distances (approximately 2.249 Å, Tables S2 and S3 in the ESI†), confirming the deviation of the Re(i) coordination sphere from the ideal octahedron.

**Fig. 5:**
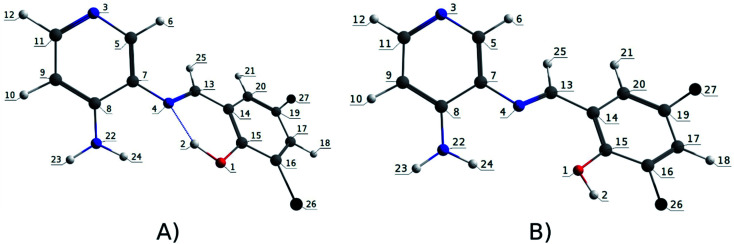
Numbering atoms of the PSB ancillary ligands. (A) PSB1, PSB2, PSB4 and PSB6; (B) PSB3 and PSB5.

In the case of R1, R2, R5 and R6, calculations were in agreement with experimental data reported for a similar *fac*-[Re(CO)_3_(4,4′-dimethyl-2,2′-bpy)((*E*)-2-((3-amino-pyridin-4-ylimino)-methyl)-4,6-di-*tert*-butylphenol)]^1+^ complex, where Re(i)–(*N*,*N*) bond distance is 2.18 (6) Å and 2.13 (5) Å, shorter than the Re(i)–PSB bond distance 2.22 (5) Å.^82^ Regarding the azomethine group (C=N), bond distances are approximately 1.307 Å (comparable to that described for a similar PSB,^87^). By contrast, R3, R7 and R9, where the IHB is not observed in their corresponding PSB (*i.e.*, PSB3 for R3 and R9, and PSB5 for R7), the distance C=N was around 1.289 Å. This result can be explained by the presence of halogen substitutions, particularly at position 6 of the phenolic ring, which enhances the electron-withdrawing effect (inductive effect^88–91^). Furthermore, the presence of the halogen could participate in forming a halogen–carbon permanent dipole, favoring the interaction with the vicinal OH group in the phenolic ring (as previously reported^92,93^) (Fig. 5), potentially affecting the photoluminescent behavior (see below).

Analysis of frequencies calculations for all complexes showed three characteristic bands associated with carbonyl groups coordinated to the rhenium core (Table S4 and Fig. S1† in the ESI). In all cases, C–O bands were found around 2033 cm^−1^, and convolution bands at 1967 cm^−1^ and 1951 cm^−1^. These results can be explained by the symmetry loss of the complexes under study. The first band (around 2033 cm^−1^) was assigned to the symmetric vibration mode of the CO. On the other hand, the convolution bands (around 1967 cm^−1^ and 1951 cm^−1^) were assigned to the antisymmetrical vibration mode of the CO (wide band). This is a consequence of the trans effect of the ancillary ligand nature, which increases the CO ligand's force constant, lowering the difference between both antisymmetric bands.^65^

The study of geometry optimization obtained by the proposed calculation protocol showed a good agreement with previously reported experimental data obtained by FTIR and UV-Vis,^34,35^ and X-ray,^82^ for similar complexes. Thus, this geometry optimization provides robust support for absorption and emission calculations, as described below.

### 3.2 Optical properties

Time-dependent density functional theory (TDDFT) calculations^94–96^ were performed to predict and characterize excited states of R1 to R10 complexes. In addition, we have carried out a study of the vertical transitions associated with the absorption of the proposed complexes. These singlet–singlet transitions occur without a change in molecular geometry (vertical) and can be mapped within the framework of the TDDFT. The assignments in terms of orbitals are intended to understand the nature of the transition, ligand-centered or charge-transferring. The theoretical calculations predicted three kinds of absorption bands in all complexes, using dichloromethane as solvent. R1, R2, and R4 (Table 1) presented a first band between 270–280 nm, a second band was located at 310–312 nm, and a third band was found at 393–398 nm. Nevertheless, we observed only two bands in R3 located around 278–279 nm and 396 nm. This difference presented by R3 could be attributed to the electron-donating effect of the phenol ring due to two fluorine substituents at positions 4 and 6 (compare R3 with R1, R2 or R4 in Fig. 1 and Table S1 in the ESI†). In addition, and as stated above, the presence of a halogen in position 6 precludes the formation of the IHB.

**Table 1:** Calculated wavelengths (nm), oscillator strengths (*f*) and corresponding transition and assignment for selected R1 to R4 complexes^a^

Complex	*λ* (nm)	*f*	Transition	Assignment*
R1	394	0.4434	H → L+1 (96%)	LLCT/MLCT(π → π*/d → π*)
	312	0.4878	H−6 → L (51%)	L_bpy_L_bpy_CT(π → π*)
	270	0.3378	H−1 → L+4 (36%)	LLCT/MLCT(π → π*/d → π*)
			H−2 → L+5 (13%)	MLCT(d → π*)
R2	393	0.4774	H → L+1 (97%)	LLCT/MLCT(π → π*/d → π*)
	311	0.3654	H−6 → L (65%)	L_bpy_L_bpy_CT(π → π*)
	280	0.4279	H−5 → L+1 (77%)	LLCT/MLCT(π → π*/d → π*)
R3	396	0.4003	H → L+1 (95%)	LLCT/MLCT(π → π*/d → π*)
	279	0.2794	H−7 → L+1 (52%)	LLCT(π → π*)
	278	0.5497	H−5 → L+1 (40%)	LLCT/MLCT(π → π*/d → π*)
R4	398	0.3026	H → L+1 (72%)	LLCT/MLCT(π → π*/d → π*)
	310	0.3191	H → L+3 (41%)	LL_bpy_CT/ML_bpy_CT(π → π*/d → π*)
			H−6 → L (41%)	L_bpy_L_bpy_CT(π → π*)
	280	0.4969	H−5 → L+1 (58%)	LLCT/MLCT(π → π*/d → π*)

a bpy: 2,2′-bpy.

The assignments and nature of the described transitions are summarized in Table 1. In R1 to R4 complexes, the calculated UV-Vis spectrum centered around 395 nm, similar to other cationic Re(i) complexes,^23,97–100^ assigned to HOMO → LUMO+1 with a mixture of π → π* and MLCT. Table 2 shows the isosurfaces of the molecular orbitals and the electron density differences maps for these first series (R1 to R4), respectively, where the calculated transitions showed that the compositions of the excitation associated with the LLCT (π → π* corresponding to PSB ancillary ligand, Scheme 1) and d → π* transitions involved the metals and PSB (Table 1).^101–103^ The most important orbitals involved in electronic transitions are qualitatively represented in Fig. S2 to S5 in the ESI.†

**Table 2:** Molecular orbitals and maps of electron density differences between the states involved in the electronic transition of absorption located at 393–398 nm for R1 to R4 (yellow: density reduction; and green: density increment due to the transition)

Complex	HOMO	LUMO+1	Density diff.
R1	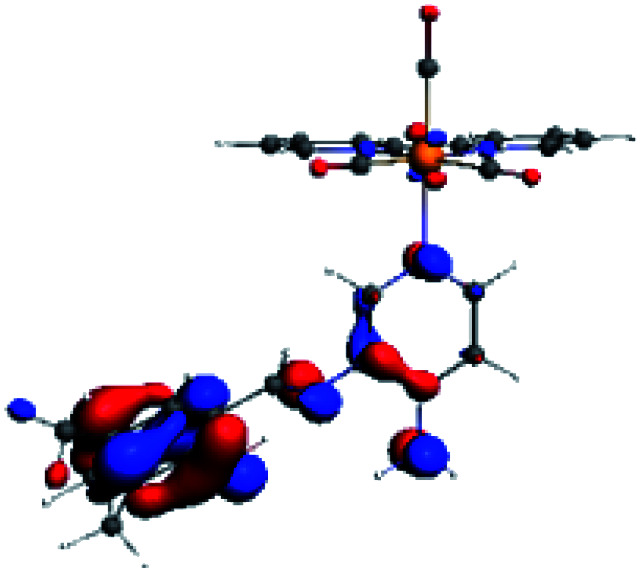	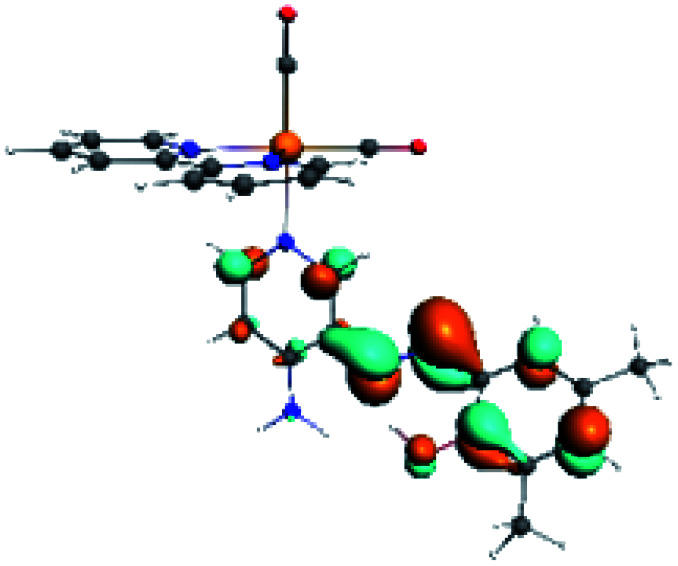	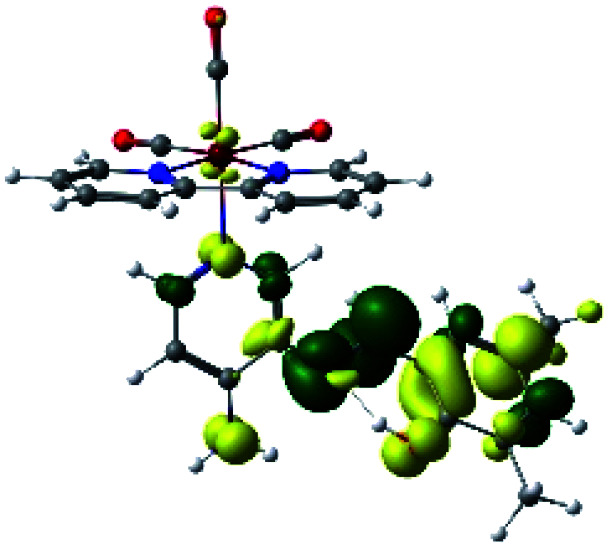
R2	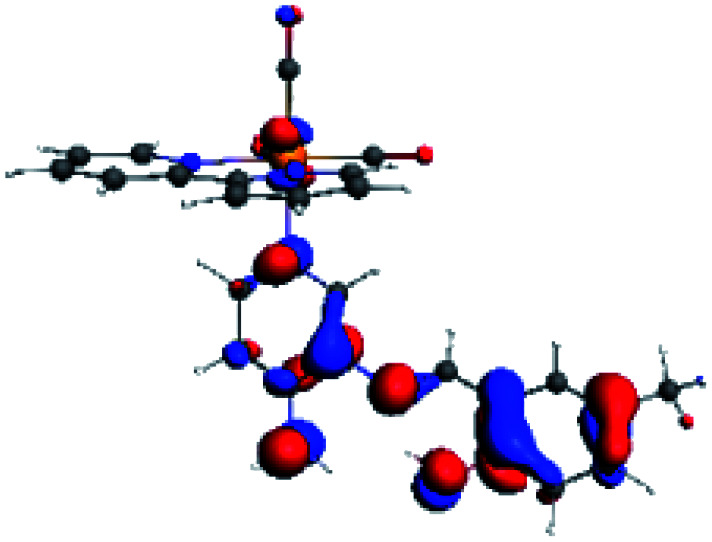	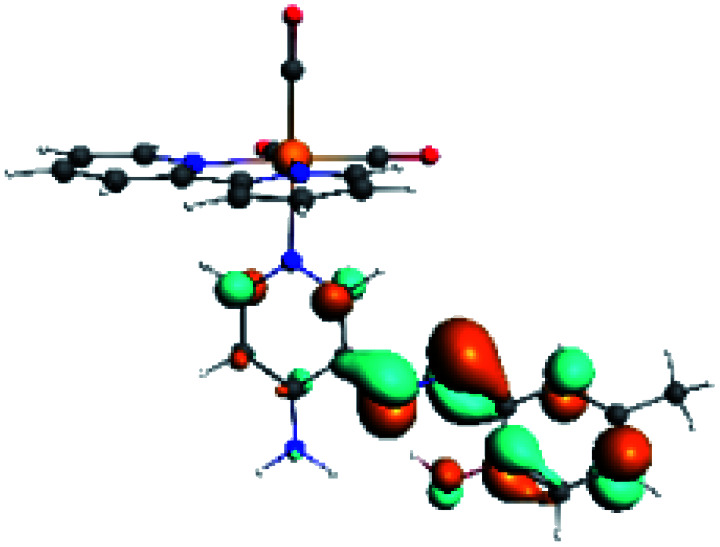	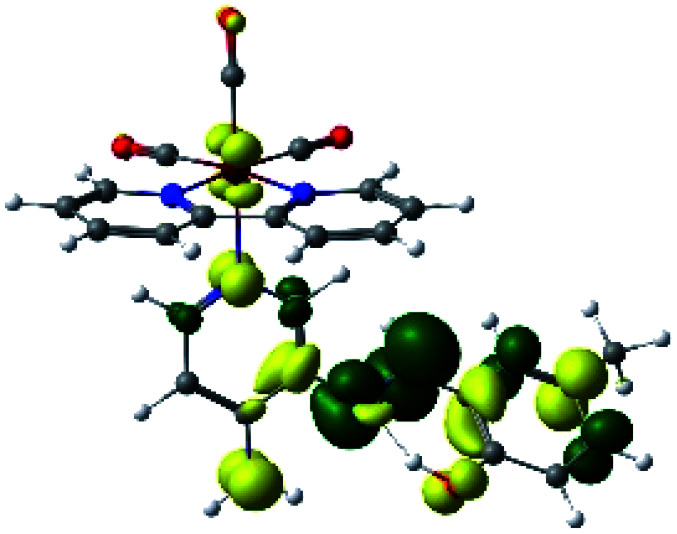
R3	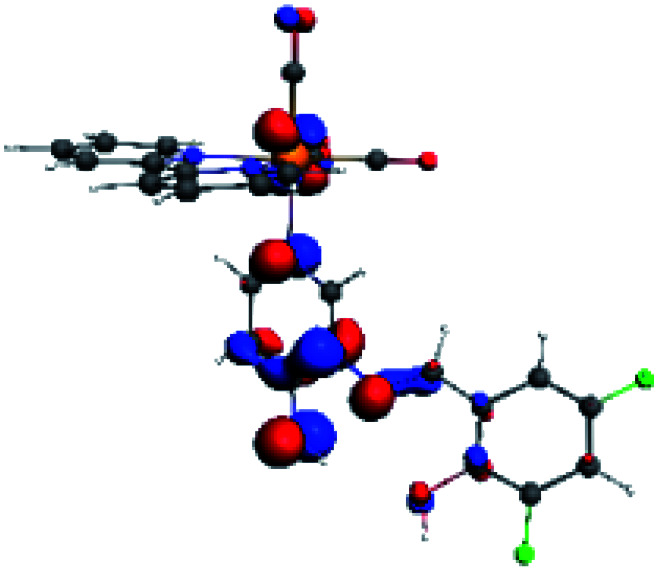	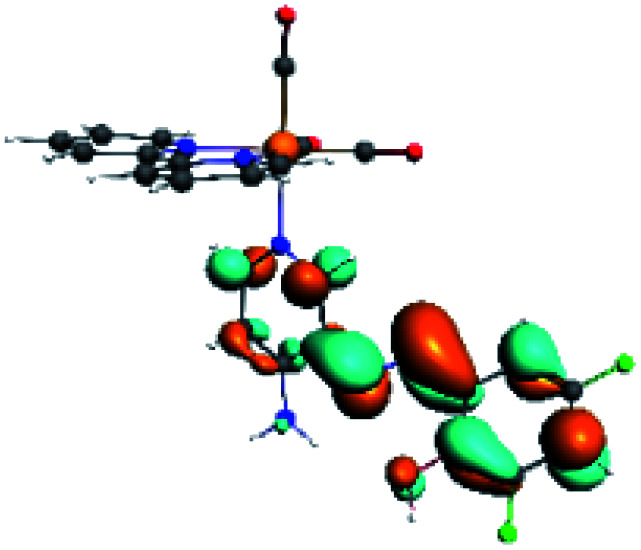	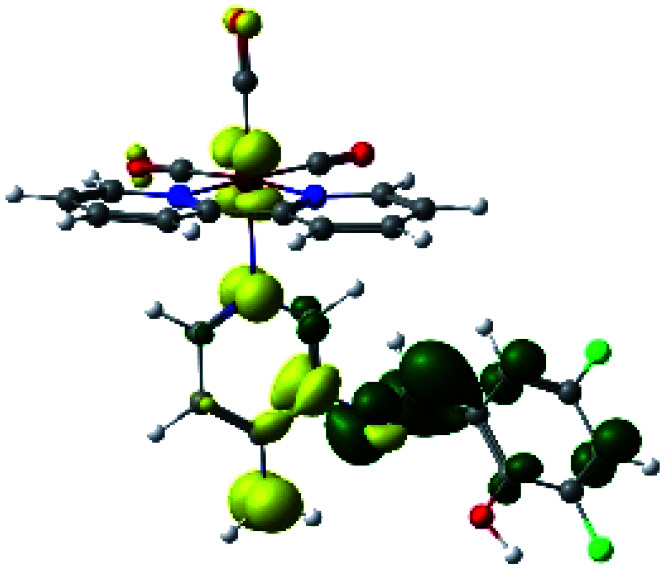
R4	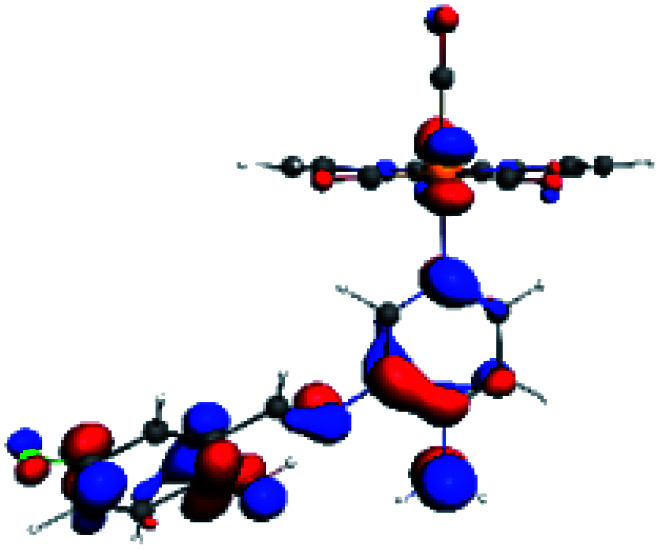	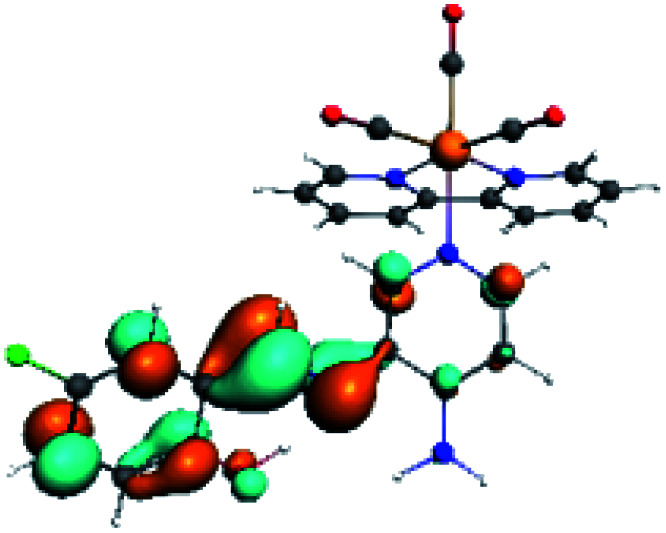	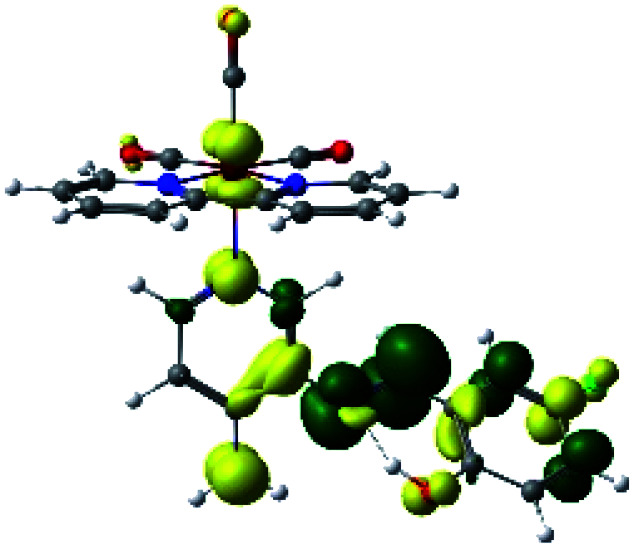

The difference electron density maps of Table 2 show that in R1, R2 and R4 the electron density is transferred to the azomethine π system (green color). In the molecular orbital analysis, it is shown that the acceptor orbital is naturally the empty π-antibonding. For R1, R2 and R4, the donor portion of the PSB (yellow color) is mainly centered at the pyridine moiety. On the other hand, the donor orbital (π) in R3 changes from the phenolic ring to the pyridinic ring, does not involve the phenolic ring given the electron-withdrawing effect of the two F atoms. This result can be attributed to a lower electron density of the phenolic ring in R3, compared with R1, R2 and R4 (Table 2). In addition, the presence of the halogen at position 6 changes the LLCT character, displacing it from the phenolic ring to the pyridine ring. Furthermore, the LUMO+1 orbital exhibits mainly a π-antibonding character centered at the azomethine group in R1 to R4 complexes (Table 2), explaining why the IHB has a significant effect on transition energies, besides the effect of the molecular rigidity on the triplet lifetime (see below).

On the other hand, R5 to R10 presented three absorption bands around 287 nm, 332 nm and 394 nm (Table 3). Regarding the transition around 394 nm, typical for cationic Re(i) complexes, as stated above, R5 to R10 showed a composition HOMO → LUMO+2, where HOMO and LUMO+2 is mainly centered among the ancillary ligand (PSB). In R5 and R6, a minority metal-centered composition was observed in comparison with R7 to R10. A second composition for 394 nm was found for R5 (HOMO → LUMO+3) and R6, R7 and R9 (HOMO → LUMO+1). Table 4 shows the isosurfaces of the molecular orbitals and the electron density differences maps for series 2 (R5 to R10) (the most important orbitals plots for R5 to R10 are qualitatively represented in Fig. S6 to S11 in the ESI†).

**Table 3:** Calculated wavelengths (nm), oscillator strengths (*f*) and corresponding transition and assignment for selected R5 to R10 complexes^a^

Complex	*λ* (nm)	*f*	Transition	Assignment*
R5	392	0.4383	H → L+2 (42%)	LLCT/LL_deeb_CT/ML_deeb_CT(π → π*/π → π*/d → π*)
			H → L+3 (31%)	
	330	0.5594	H−7 → L (52%)	L_deeb_L_deeb_CT(π → π*)
	283	0.2532	H−5 → L+2 (63%)	LLCT/LL_deeb_CT/ML_deeb_CT(π → π*/π → π*/d → π*)
R6	395	0.2921	H → L+2 (46%)	LLCT/MLCT(π → π*/d → π*)
			H → L+1 (38%)	LL_deeb_CT/ML_deeb_CT(π → π*/d → π*)
	333	0.4156	H−6 → L (83%)	L_deeb_L_deeb_CT(π → π*)
	279	0.3421	H−5 → L+2 (74%)	LLCT/MLCT(π → π*/d → π*)
R7	388	0.3695	H → L+2 (47%)	LLCT/MLCT(π → π*/d → π*)
			H → L+1 (46%)	LL_deeb_CT/ML_deeb_CT(π → π*/d → π*)
	332	0.5483	H−7 → L (49%)	L_deeb_L_deeb_CT(π → π*)
			H−3 → L+1 (42%)	ML_deeb_CT(d → π*)
	286	0.2954	H−6 → L+2 (61%)	LLCT/MLCT(π → π*/d → π*)
R8	400	0.386	H → L+2 (73%)	LLCT/MLCT(π → π*/d → π*)
	332	0.4896	H−6 → L (45%)	L_deeb_L_deeb_CT(π → π*)
			H−2 → L+1 (34%)	ML_deeb_CT(d → π*)
	318	0.2338	H−3 → L+2 (40%)	LLCT/MLCT(π → π*/d → π*)
			H−3 → L+1 (38%)	ML_deeb_CT(d → π*)
R9	387	0.3142	H → L+1 (48%)	LL_deeb_CT/ML_deeb_CT(π → π*/d → π*)
			H → L+2 (43%)	LLCT/MLCT(π → π*/d → π*)
	332	0.5538	H−7 → L (55%)	L_deeb_L_deeb_CT(π → π*)
	275	0.277	H−5 → L+2 (45%)	LLCT/MLCT(π → π*/d → π*)
R10	401	0.4929	H → L+2 (83%)	LLCT/MLCT(π → π*/d → π*)
	333	0.4639	H−6 → L (81%)	L_deeb_L_deeb_CT(π → π*)
	281	0.3865	H−5 → L+2 (35%)	LLCT/MLCT(π → π*/d → π*)
			H−5 → L+1 (27%)	LL_deeb_CT/ML_deeb_CT(π → π*/d → π*)

a deeb: 4,4′-bis(ethoxycarbonyl)-2,2′-bpy.

**Table 4:** Molecular orbitals and maps of electron density differences between the states involved in the electronic transition of absorption located at 387–401 nm for R5 to R10 (yellow: density reduction; and green: density increment due to the transition)

Complex	HOMO	LUMO+3	LUMO+2	Density diff.
R5	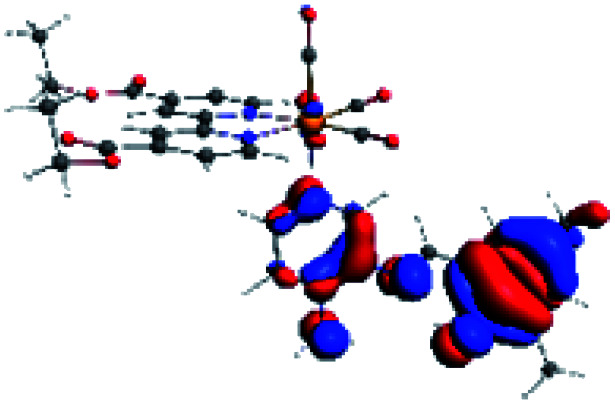	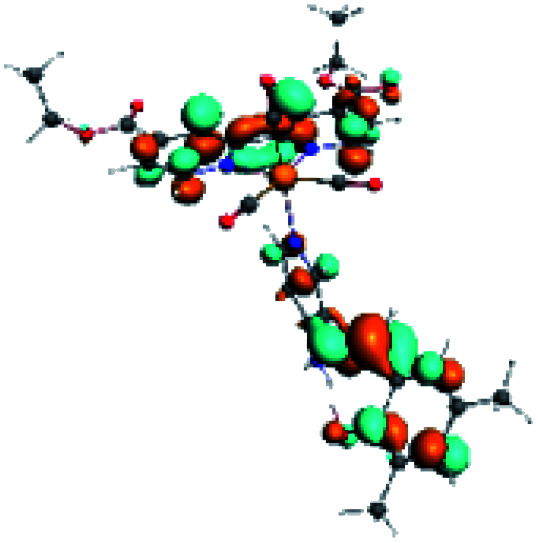	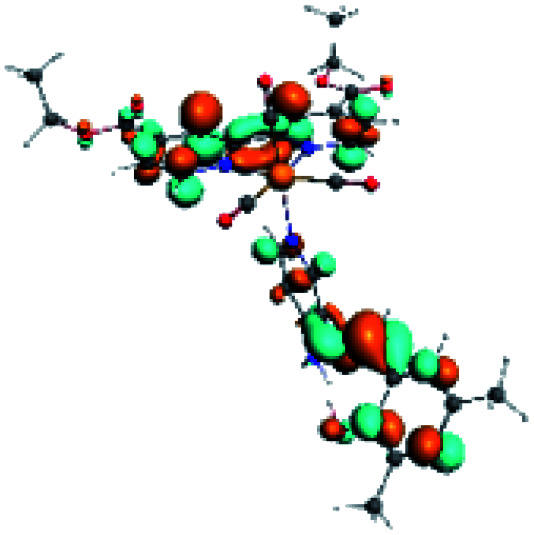	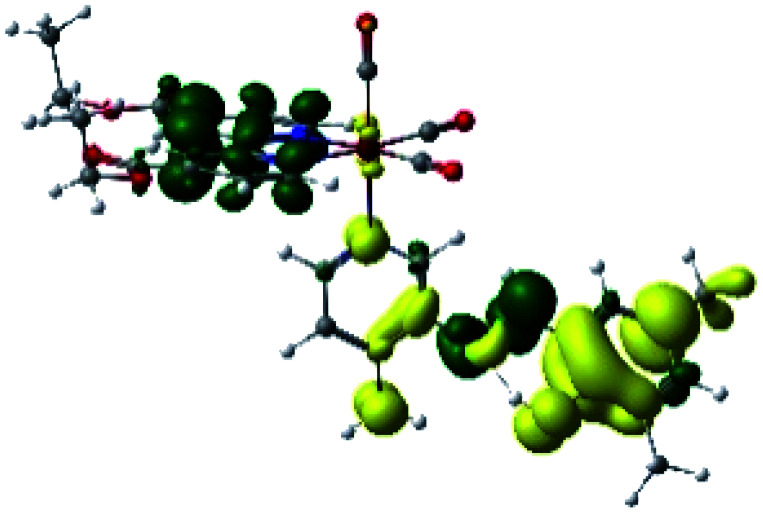
R6	HOMO	LUMO+1	LUMO+2	Density diff.
	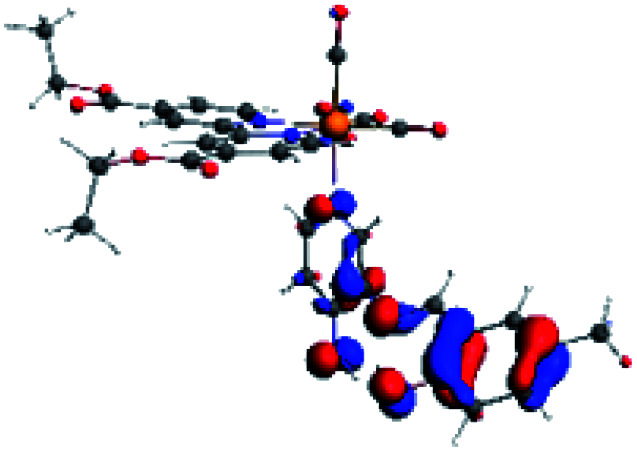	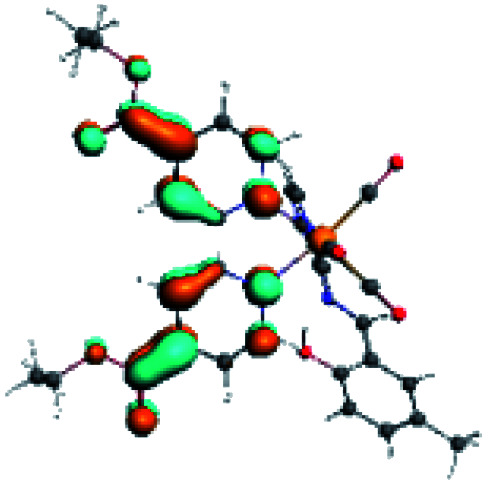	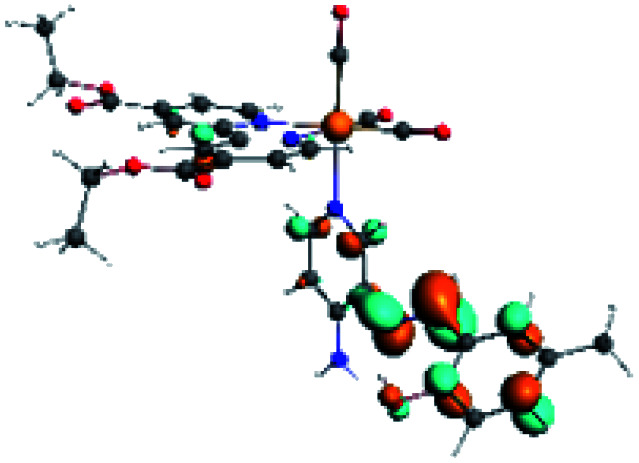	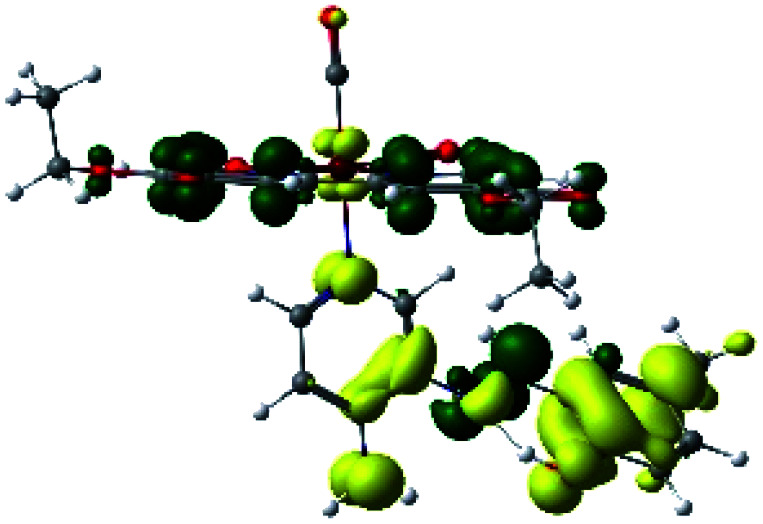
R7	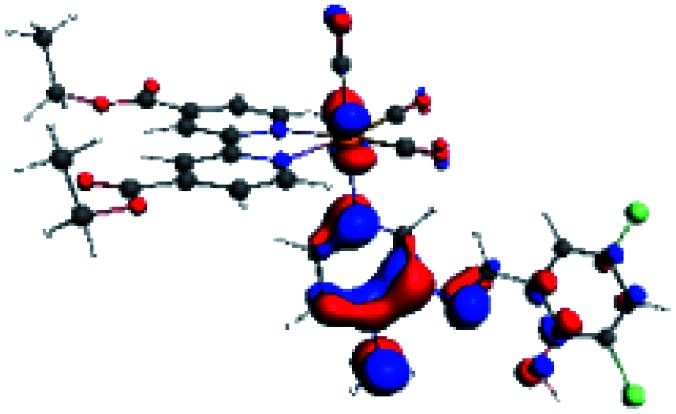	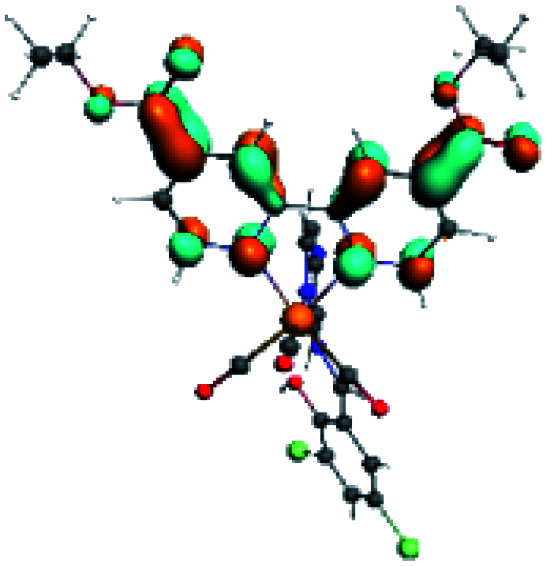	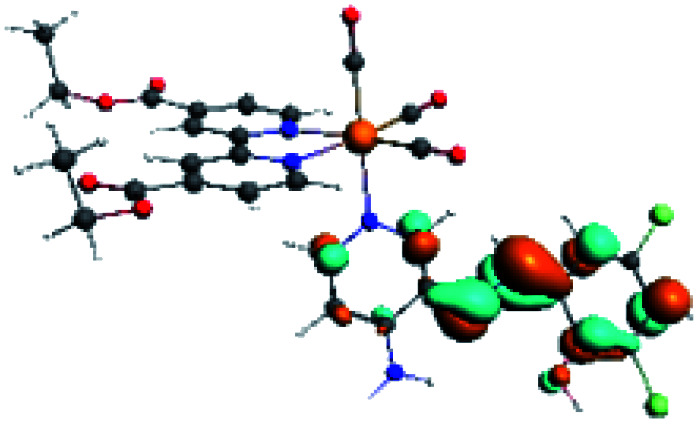	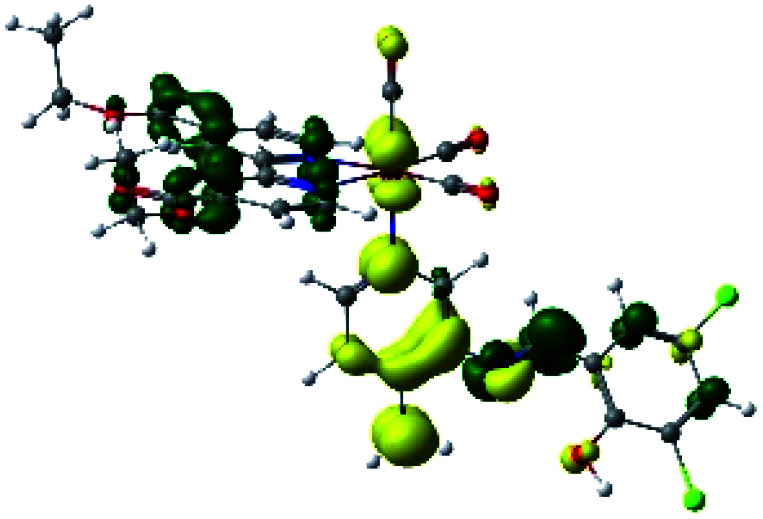
R8	HOMO		LUMO+2	Density diff.
	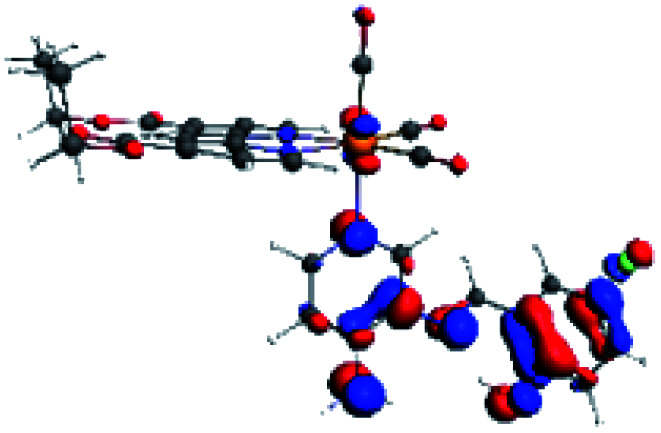		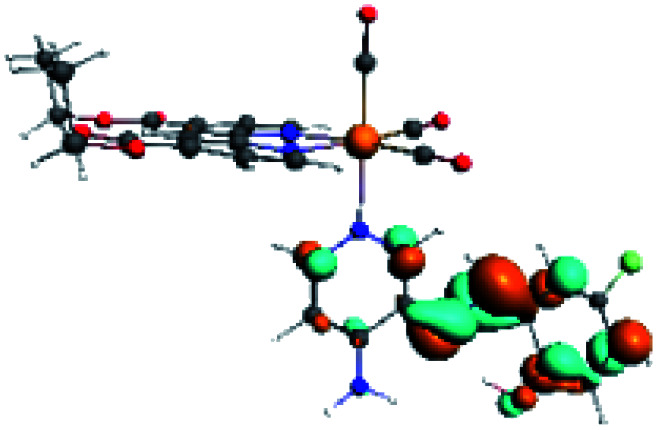	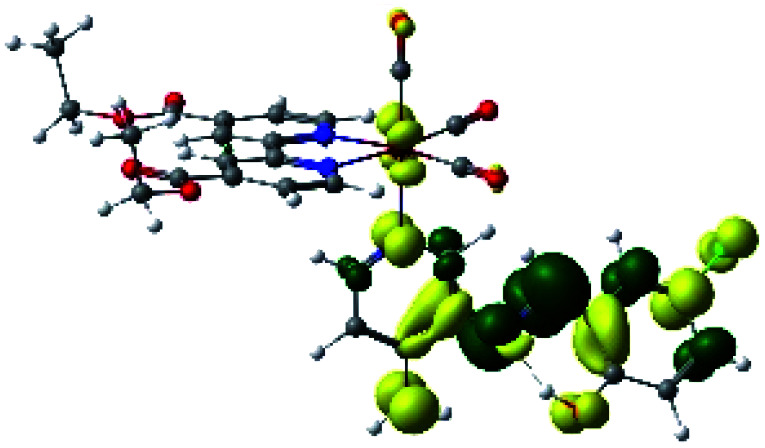
R9	HOMO	LUMO+1	LUMO+2	Density diff.
	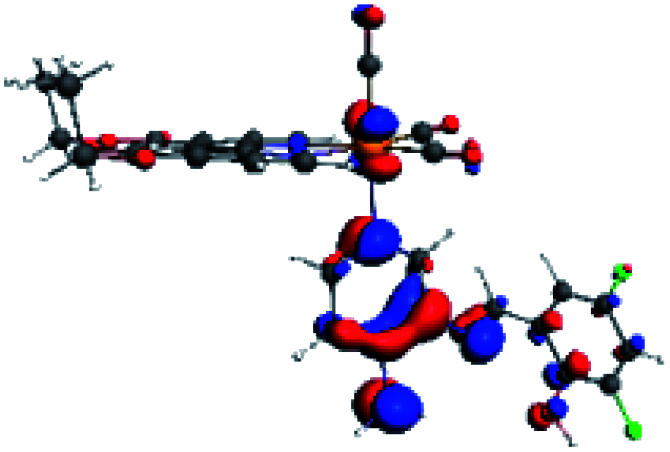	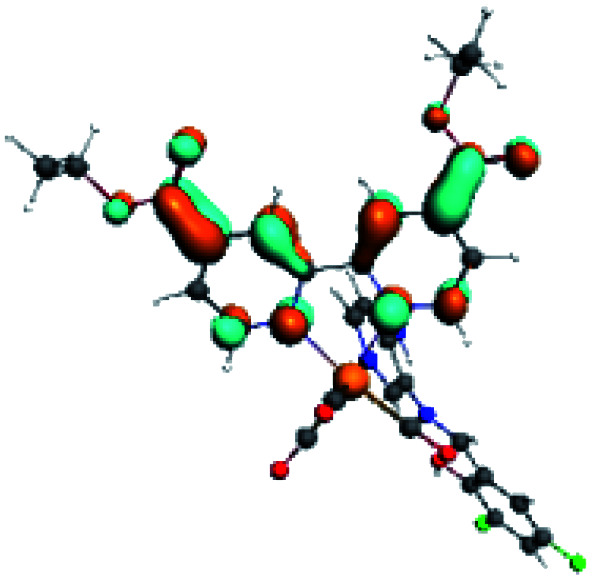	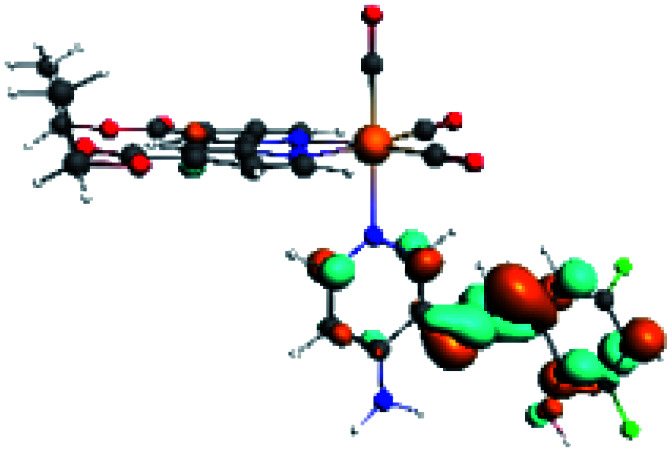	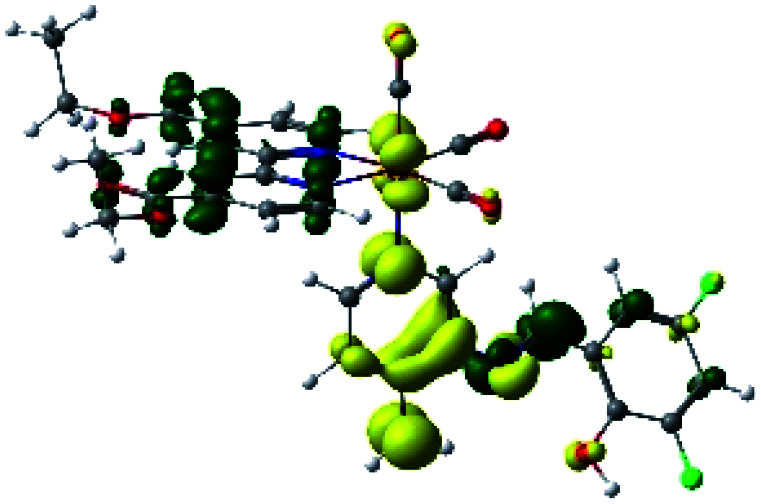
R10	HOMO		LUMO+2	Density diff.
	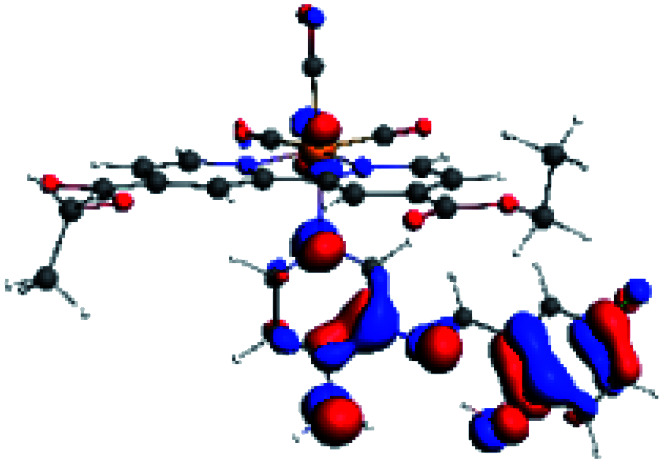		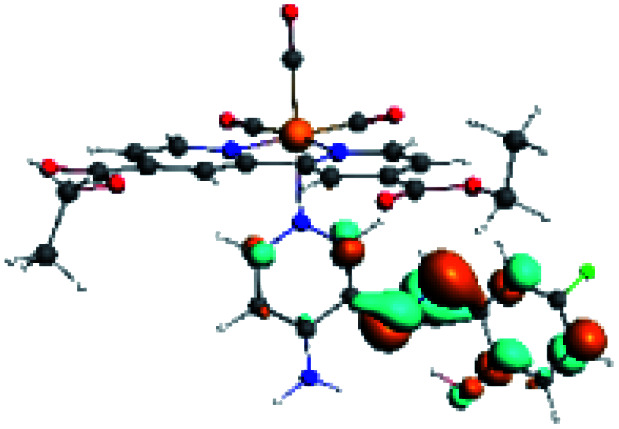	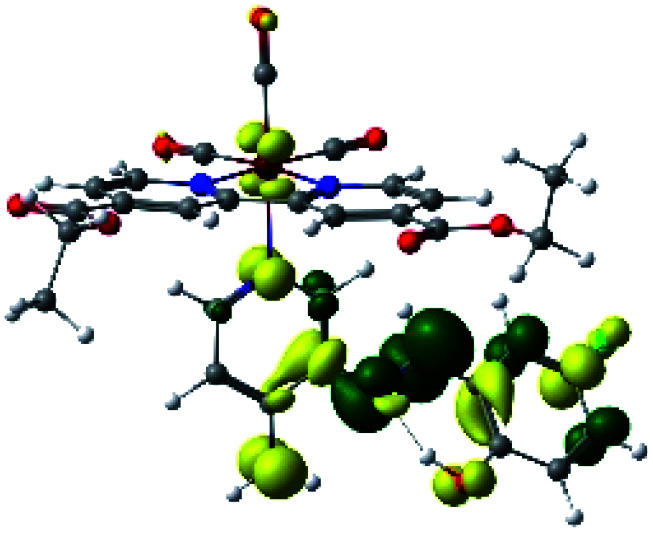

In addition, the HOMO orbital for R7 and R9 is more localized in the phenolic ring due to a lower electron density, compared with the same ring in R5, R6, R8 and R10 (Table 4). The LUMO+2 orbital showed mainly a π-antibonding character centered at the azomethine group in R5 to R10 complexes (Table 4). Thus, as well as with R3, the IHB has an important effect on the transition energies for R7 and R9 complexes.

The experimental absorption of precursors of the complexes analyzed in the present study, which have Br instead of PSB, was already reported. The precursor for series 1 complexes (*i.e.*, *fac*-Re(i)(CO)_3_(2,2′-bpy)Br) showed a *λ*_max_ = 383 nm in acetonitrile;^34,61^ whereas the precursor for series 2 (*i.e.*, *fac*-Re(i)(CO)_3_(4,4′-bis(ethoxycarbonyl)-2,2′-bpy)Br) exhibited a *λ*_max_ = 419 nm in dichloromethane or acetonitrile.^35,36^ When Br was replaced by a PSB harboring an IHB (*i.e.*, (*E*)-2-(((4-aminopyridin-3-yl)imino)methyl)-4,6-di-*tert*-butylphenol), similar to those analyzed in this manuscript, an experimental blue-shifted absorption was observed compared with their respective precursors, which was attributed to the presence of the ancillary ligand (*i.e.*, *fac*-[Re(i)(CO)_3_(2,2′-bpy)((*E*)-2-(((4-aminopyridin-3-yl)imino)methyl)-4,6-di-*tert*-butylphenol)]^1+^ [acetonitrile, *λ*_max_ = 361 nm] and *fac*-[Re(i)(CO)_3_(4,4′-bis(ethoxycarbonyl)-2,2′-bpy)((*E*)-2-(((4-aminopyridin-3-yl)imino)methyl)-4,6-di-*tert*-butylphenol)]^1+^ [dichloromethane or acetonitrile, *λ*_max_ = 361 nm].^34,35^ The theoretical protocol used in the present manuscript, considered a blue-shift aborsorption of complexes harboring this kind of PSBs with respect to their corresponding precursors to perform calculations.

### 3.3 Theoretical study of emission

We have considered, as usually happens, that the emissions occur after a process of geometric reorganization in the triplet state (much faster in general than the radiative mechanisms); thus, the geometry of this state has been optimized, and the SOC-TDDFT calculations have been performed considering this new geometry. Then, we first analyzed the geometry optimizations of the singlet and triplet states for all complexes (Tables S5 and S6 in the ESI†).

In the singlet state, the O–H bond distance (Table S1 in the ESI†) were observed around 1.008 Å for PSB1, PSB2, PSB4 and PSB6; and, where the IHB is not present, this distance was shorter (≈0.980 Å) (*i.e.*, for PSB3 and PSB5). On the other hand, the H⋯N (IHB) bond distance was observed around 1.696 Å for PSB1, PSB2, PSB4 and PSB6; whereas the H⋯F (PSB3) and H⋯Cl (PSB5) bond distances were obtained at 2.127 Å and 2.268 Å, respectively. For R3, R7 and R9 we observed an interaction between the hydrogen of the –OH group in the phenolic ring and neighbored halogen in the same ring (PSB3 or PSB5). The C_14_C_15_OH bond angle in PSB1, PSB2, PSB4 and PSB6 (S_0_ state) were observed around −0.30°; for PSB3 and PSB5, in the corresponding R3, R7 and R9, appeared at −179.37° (Tables S5 and S6 in the ESI†; and numbering atoms in Fig. 5). In R1, R2, R4, R5, R6, R8 and R10, the formation of IHB was observed; therefore, the distances and bond angles showed the structural differences indicated above, compared to R3, R7 and R9. The geometric parameters of the triplet state presented a similar behavior when compared with the R1 to R10 complexes.

In a previous study, the photoluminescence of seven *fac*-Re(i) tricarbonyl complexes showed similar maximum emission wavelengths, regardless of the ancillary ligand tested (including pyridine, imidazole, or triphenylphosphine).^17^ By contrast, we found differences in the emission of R1, R2, R4, R5, R6, R8, and R10 with R3, R7, and R9, showing that, in our case, the ancillary ligand does exert an impact on the photoluminescent properties. This result can be mainly attributed to the presence of IHB in the corresponding ancillary ligand, as described above, remarking the importance of this intramolecular interaction regarding the emission behavior.

Regarding the geometry of R3, R7 and R9, we found differences between the singlet S_0_ and triplet state T_1_ in each case, involving changes in bond distances and angles. These differences between the states can be attributed to a structural relaxation from the lowest triplet excited state (T_1_) to the ground state (S_0_). By contrast, the presence of the IHB precludes this relaxation; accordingly, R1, R2, R4, R5, R6, R8 and R10, only show slight geometrical differences between the singlet S_0_ and triplet state T_1_.

In order to calculate the emission lifetime from the T_1_ state, a SOC-TDDFT calculation was done on the triplet from the electronic singlet ground state on that geometry.^104^ Typically, the three lowest sub-states of the spin–orbit coupling TDDFT calculation are important, since they resemble the three sub-states in a triplet state.^105,106^ There will be a slight energy difference between the three sub-states because of spin–orbit coupling, the so-called zero-field splitting (ZFS). Based on the described methodology, the emission calculated for R1, R2, R4, R5, R6, R8 and R10 was observed in 622–673 nm (Table 5). Experimental data previously obtained for *fac*-[Re(i)(CO)_3_(2,2′-bpy)((*E*)-2-(((4-aminopyridin-3-yl)imino)methyl)-4,6-di-*tert*-butylphenol)]^1+^, a complex that only differs from R1 in the substitution at positions 4 and 6 in the phenolic ring, presenting *tert*-butyls instead methyls, shows *λ*_ex_ = 366 nm and *λ*_em_ = 610 nm in acetonitrile.^34^ On the other hand, emission experimental data of *fac*-[Re(i)(CO)_3_(4,4′-bis(ethoxycarbonyl)-2,2′-bpy)((*E*)-2-(((4-aminopyridin-3-yl)imino)methyl)-4,6-di-*tert*-butylphenol)]^1+^ which is similar to R5, but presents *tert*-butyls instead of methyls at positions 4 and 6 in the phenolic ring, exhibit *λ*_ex_ = 365 nm, *λ*_em_ = 660 nm in dichloromethane.^35^ These experimental data support the calculations presented for R1, R2, R4, R5, R6, R8 and R10 (Fig. S12 and S13 in the ESI†).

**Table 5:** Calculated emission wavelengths (*λ*), emission lifetimes (*τ*_r_), ZFS (cm^−1^) and dominant emission character of R1 to R10

Complex	*λ* (nm)	*τ*_r_ (s)	ZFS (cm^−1^)	Transition	Assignment^a^
R1	643	5.04 × 10^−2^	0.08	L+1 → H	LLCT
R2	654	7.40 × 10^−2^	0.08	L+1 → H	LLCT
R3	869	2.76 × 10^−1^	0.00	L+1 → H	LLCT
	540	4.36 × 10^−4^	7.90	L → H (T_2_)	L_bpy_LCT
R4	673	5.59 × 10^−2^	0.00	L+1 → H	LLCT
R5	625	1.36 × 10^−3^	13.87	L+1 → H	L_deeb_LCT
R6	625	3.15 × 10^−3^	0.40	L+1 → H	LLCT
				L+2 → H	L_deeb_LCT
R7	726	3.64 × 10^−2^	0.40	L+1 → H	LLCT
	641	3.33 × 10^−3^	12.14	L → H (T_2_)	L_deeb_LCT
R8	622	2.23 × 10^−2^	1.05	L+1 → H	LLCT
				L+2 → H	L_deeb_LCT
R9	771	8.90 × 10^−2^	0.16	L+1 → H	LLCT
	649	9.77 × 10^−4^	7.78	L → H (T_2_)	L_deeb_LCT
R10	637	2.53 × 10^−2^	0.08	L+1 → H	LLCT
				L+2 → H	L_deeb_LCT

a bpy: 2,2′-bpy; deeb:4,4′-bis(ethoxycarbonyl)-2,2′-bpy.

For R3, R7 and R9, the predicted emission was identified at 869 nm, 726 nm and 771 nm, respectively (Table 5). The lowest triplet excited states in R3, R7, R9 are mainly due to π → π* transitions of the PSB (di-halogenated ligand in these cases) (Fig. S12 and S13 in the ESI†). The lowest triplet excited state energy falls into the NIR region. This result could be explained by the effect of halide substituents at positions 4 and 6 in the phenolic ring, with a reduction of the excited energy as a consequence and, on the other hand, the absence of the IHB, which substantially reduces the vibronic relaxation, diminishing the emission probability. In general, complexes exhibiting high wavelength emission (*i.e.*, close to the NIR) are less desirable for confocal microscopy due to a lower quantum efficiency (*i.e.*, low detection capability), potentially producing dimmer images.^27,100^ However, these complexes could be more suitable for other purposes, such as OLEDs,^23,107,108^ or photosensitizers for singlet oxygen generation,^109^ among other applications.

In addition, we performed an exhaustive scan for R3, R7 and R9 to find other excited triplet states. In all these cases, we found a possible emission (in the range 540–649 nm) with a sufficiently long lifetime (4.36 × 10^−4^ to 3.33 × 10^−3^ s) from a second excited triplet state (Table 5, Fig. S12 and S13 in the ESI†). Furthermore, the low intensity of the observed emission of R3, R7 and R9 could be explained by a simultaneous non-radiative deactivation of the second triplet, which competes to reach the first excited state due to the lack of the IHB in the respective PSB, as discussed above. A similar phenomenon was experimentally observed with *fac*-Re(CO)_3_(4,5-diazafluoren-9-one)Br complex,^27^ showing that the theoretical methodology used in this work is accurate for this kind of analysis.

For all the complexes (R1 to R10), the emission can be attributed to a ligand to ligand charge transfer transition involving a π* orbital (LUMO+1 and LUMO+2) located at the (*N*,*N*) or the PSB ligand, where the π orbitals (HOMO) were found in the corresponding PSB ancillary ligands (Fig. S12 and S13 in the ESI†). Finally, in all cases, the emission mechanism suggests that, after absorption, a triplet excited state is reached, attributed to ^3^LLCT. This assignment was supported by the calculated ZFS^43^ (Table 5).

**Fig. 6:**
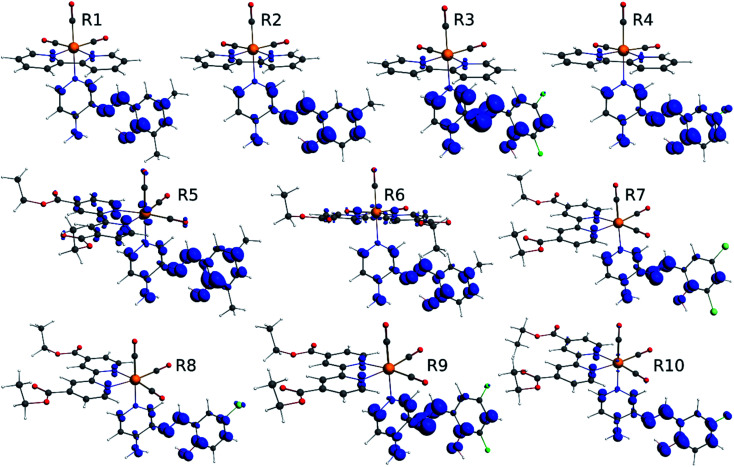
Spin density plots of the optimized triplet states for R1 to R10.

In order to confirm the nature of the emissive electronic state, the spin density distributions were obtained for all the complexes. As shown in Fig. 6, the spin density was predominantly localized on the ancillary ligand PSB, with a minimal contribution of the nitrogen atoms (in the denitrogenated *N*,*N* ligands). Thus, the spin density observed in Fig. 6 is consistent with the predominant emission character of the corresponding calculated transitions, with the ligand-centered ZFS classification (^3^LC) for all the complexes, except for R5. R5 classification is ^3^LC /^3^MLCT, where the spin density is distributed among the metal (*N*,*N*), and PSB.

In summary, for R1, R2, R5, R6, R8 and R10, the photophysical behavior was explained by the presence of the IHB, which stabilizes the planar *E* conformation. On the other hand, in the case of R3, R7 and R9, the interaction C–halogen⋯H–O might contribute to the photophysical response by the inductive effects of the halogen at position 6, as stated above. Thus, in this work, we postulate that the IHB is important regarding the emission wavelength since a potential disruption of this interaction could promote the emission shift from the visible range to the NIR range. In this context, it is possible to speculate that the IHB could be disrupted in protic solvents such as in the biological environment. Nevertheless, it has been reported that, in similar complexes, the IHB seems to resist the presence of a protic solvent. For instance, a previous study of a Re(i) tricarbonyl complexes harboring a similar pyridine Schiff base harboring IHB [(*E*)-2((3-amino-pyridin-4-ylimino)-methyl)-4,6-diterbutylphenol] to those shown in the present manuscript, showed no significant differences regarding their optical or luminescent properties when diluted in dichloromethane (*ε* = 8.93) or acetonitrile (*ε* = 37.5).^34,35^ In addition, it was reported that, in studies involving only the [(*E*)-2((3-amino-pyridin-4-ylimino)-methyl)-4,6-diterbutylphenol], the H-bond stability is independent of solvent polarity;^39^ this same result was also observed with other similar Schiff bases harboring IHB.^40^ Furthermore, confocal microscopy studies performed with these complexes, whose stock was diluted in DMSO, allowed obtaining successful images.^37^ Interestingly, in that study, the confocal microscope was set with laser excitation at 405 nm and emission of 555 to 625 nm.^37^ This emission is in the visible range, not in the NIR, as expected for complexes harboring the IHB. All these results argue the high stability of the IHB, even in the presence of protic solvents or in cellular environments, as previously stated.^37^ Finally, if indeed the IHB were to break in biological conditions, the complexes would exhibit different emissions dependent on the cellular microenvironment, *e.g.*, cytoplasm (protic environment) or cell membranes (aprotic environments), potentially generating a differential staining. However, the studied complexes must be mandatorily tested to elucidate their behavior with cells.

## 4. Conclusions

Relativistic DFT and TDDFT methods were used to investigate the effect on electronic structures in two series of Re(i) tricarbonyl complexes. In each case, the electronic structure analysis showed that R3, R7 and R9 emit in the NIR region with a low emission probability from the second excited triplet state. On the other hand, R1, R2, R4, R5, R6, R8 and R10 presented emissions in the range of 622–673 nm from a ^3^LLCT electronic state. According to our results, an IHB in the PSB ancillary ligand is crucial to modulating photophysical properties. In this sense, the presence of a halogen at position 6 in the phenolic ring precludes the formation of the IHB, affecting the emission wavelength and lifetime. These results reinforce the concept of structure–functionality relationship in this kind of complex.

The increasing development of biotechnological applications based on microorganisms (especially walled-cell models such as bacteria and fungi), including the need for their identification and diagnosis and protein staining, emphasizes the need for systematic research of new cationic Re(i) tricarbonyl complexes. This study could contribute to predicting properties of Re(i) tricarbonyl complexes to be synthesized, considering the desired emission properties for biological applications.

## Conflicts of interest

There are no conflicts to declare.

## Supplementary Material

RA-011-D1RA05737E-s001
