# Correction: The role of substituted pyridine Schiff bases as ancillary ligands in the optical properties of a new series of *fac*-rhenium(i) tricarbonyl complexes: a theoretical view

**DOI:** 10.1039/d1ra90173g

**Published:** 2021-12-01

**Authors:** Rosaly Morales-Guevara, Juan A. Fuentes, Dayán Paez-Hernández, Alexander Carreño

**Affiliations:** Universidad Andres Bello, Programa de Doctorado en Físicoquímica Molecular, Facultad de Ciencias Exactas Santiago Chile alexander.carreno@unab.cl dayan.paez@unab.cl; Laboratory of Organometallic Synthesis, Center of Applied NanoSciences (CANS), Facultad de Ciencias Exactas, Universidad Andres Bello República 330 Santiago Chile alexander.carreno@unab.cl dayan.paez@unab.cl; Laboratorio de Genética y Patogénesis Bacteriana, Facultad de Ciencias de la Vida, Universidad Andres Bello República 330 Santiago Chile

## Abstract

Correction for ‘The role of substituted pyridine Schiff bases as ancillary ligands in the optical properties of a new series of *fac*-rhenium(i) tricarbonyl complexes: a theoretical view’ by Rosaly Morales-Guevara *et al.*, *RSC Adv.*, 2021, **11**, 37181–37193, DOI: 10.1039/D1RA05737E.

The authors regret that the email address of one of the corresponding authors (Dayán Paez-Hernández) was shown incorrectly in the original manuscript. The corrected email is as follows: dayan.paez@unab.cl.

The Royal Society of Chemistry apologises for these errors and any consequent inconvenience to authors and readers.

## Supplementary Material

