# Supplementary material for: The role of substituted pyridine Schiff bases as ancillary ligands in the optical properties of a new series of fac-rhenium(i) tricarbonyl complexes: a theoretical view
Source: RSC Adv. 2021 Nov 18;11(59):37181–93. doi: 10.1039/d1ra05737e (PMC9043815; doi:10.1039/d1ra05737e)
Supplement: RA-011-D1RA05737E-s001 [file RA-011-D1RA05737E-s001.pdf]

Electronic Supplementary Material (ESI) for RSC Advances.
This journal is © The Royal Society of Chemistry 2021

Supplementary Material

The role of the substituted pyridine Schiff bases as ancillary ligands in the optical properties of a new series of *fac*-rhenium(I) tricarbonyl complexes: A theoretical view

Rosaly Morales-Guevara,[1,2] Juan A. Fuentes,[3] Dayán Paez-Hernández,[1,2*] Alexander Carreño[1,2*]

[1]Universidad Andres Bello, Programa de Doctorado en Físicoquímica Molecular, Facultad de Ciencias Exactas, Santiago, Chile.

[2]Center of Applied Nano Sciences (CANS), Facultad de Ciencias Exactas, Universidad Andres Bello, República 330, Santiago, Chile.

[3]Laboratorio de Genética y Patogénesis Bacteriana, Facultad de Ciencias de la Vida, Universidad Andrés Bello, República 330, Santiago, Chile.

*Corresponding authors: alexander.carreno@unab.cl; d.paez@unab.cl

Section 1: Supplementary Tables

Table S1. Re(I) tricarbonyl complexes and Schiff bases (**PSB**) analyzed in this study.

Re(I) Complex	(N,N)	Pyridine Schiff Base	Structure Pyridine Schiff Base
**R1**	2,2'-bpy	(*E*)-2-(((4-aminopyridin-3-yl)imino)methyl)-4,6-di-methylphenol	PSB1 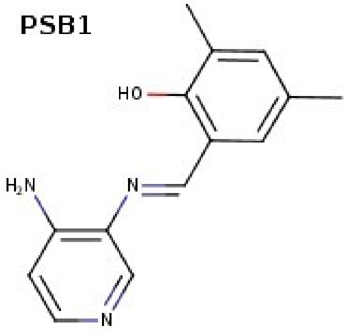
**R2**	2,2'-bpy	(*E*)-2-(((4-aminopyridin-3-yl)imino)methyl)-4-methylphenol	PSB2 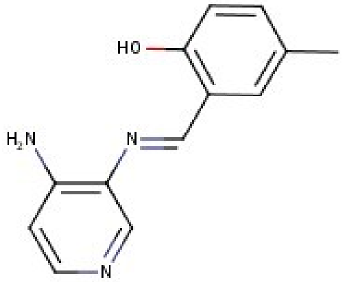
**R3**	2,2'-bpy	(*E*)-2-(((4-aminopyridin-3-yl)imino)methyl)-4,6-di-fluorophenol	PSB3 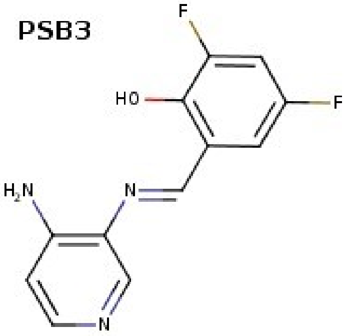
**R4**	2,2'-bpy	(*E*)-2-(((4-aminopyridin-3-yl)imino)methyl)-4-fluorophenol	PSB4 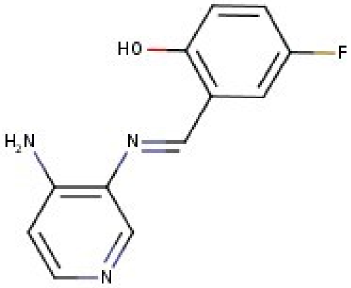

**Re(I) Complex**	**(N,N)**	**Pyridine Schiff Base**	**Structure Pyridine Schiff Base**
**R5**	4,4´-bis(ethoxycarbonyl)-2,2´-bpy	(*E*)-2-(((4-aminopyridin-3-yl)imino)methyl)-4,6-di-methylphenol	
**R6**	4,4´-bis(ethoxycarbonyl)-2,2´-bpy	(*E*)-2-(((4-aminopyridin-3-yl)imino)methyl)-4-methylphenol	
**R7**	4,4´-bis(ethoxycarbonyl)-2,2´-bpy	(*E*)-2-(((4-aminopyridin-3-yl)imino)methyl)-4,6-di-chlorophenol	PSB5 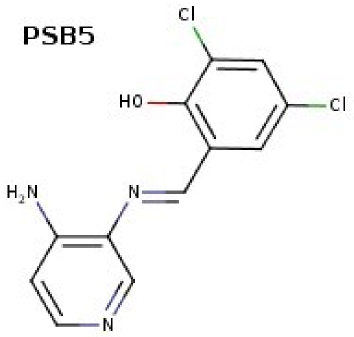
**R8**	4,4´-bis(ethoxycarbonyl)-2,2´-bpy	(*E*)-2-(((4-aminopyridin-3-yl)imino)methyl)-4-chlorophenol	PSB6 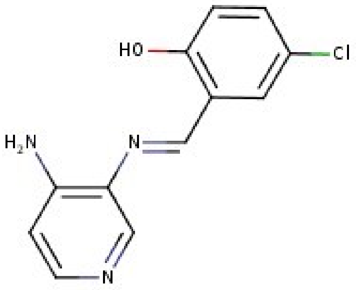

Re(I) Complex	(N,N)	Pyridine Schiff Base	Structure Pyridine Schiff Base
**R9**	4,4´-bis(ethoxycarbonyl)-2,2´-bpy	(*E*)-2-(((4-aminopyridin-3-yl)imino)methyl)-4,6-di-fluorophenol	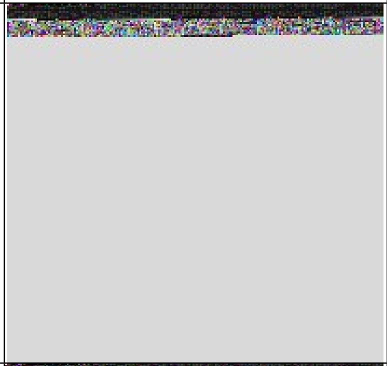
**R10**	4,4´-bis(ethoxycarbonyl)-2,2´-bpy	(*E*)-2-(((4-aminopyridin-3-yl)imino)methyl)-4-fluorophenol	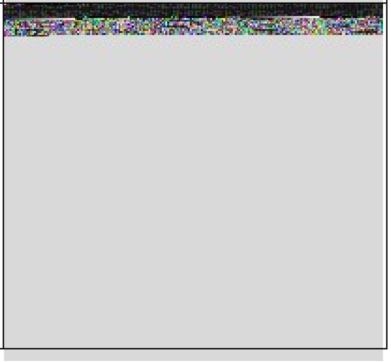

Table S2. Optimized geometry parameters for **R1** to **R4** in the ground state.

Parameters	R1	R2	R3	R4
**Bond length (Å)**				
**Re-N (L)**	2.249	2.246	2.249	2.248
**Re-N(dinitrogenated)***	2.195	2.193	2.193	2.193
**Re-CO(axial)**	1.938	1.938	1.939	1.939
**Re-CO(eq)***	1.932	1.932	1.933	1.933
**C=O (axial)**	1.159	1.159	1.159	1.159
**C=O (eq)***	1.162	1.162	1.162	1.162
**N=C(azomethine)**	1.309	1.307	1.290	1.305
**Bond angle (°)**				
**N-Re-N(L)**	86.00	85.26	85.41	86.34
**N-Re-N**	74.31	74.31	74.36	74.37
**N-Re-C(eq)**	171.82	171.75	171.61	172.12
**C(axial)-Re-N(L)**	179.62	179.19	179.57	179.51
**C(eq)-Re-C(axial)**	89.66	89.49	89.58	89.34

**average values of two bond distances*

Table S3. Optimized geometry parameters for **R5** to **R10** in the ground state.

Parameters	R5	R6	R7	R8	R9	R10
**Bond length (Å)**						
**Re-N (L)**	2.250	2.250	2.246	2.247	2.247	2.251
**Re-N(dinitrogenated)***	2.194	2.194	2.191	2.191	2.193	2.191
**Re-CO(axial)**	1.938	1.938	1.939	1.939	1.939	1.939
**Re-CO(eq)***	1.932	1.932	1.933	1.933	1.932	1.933
**C=O (axial)**	1.159	1.159	1.159	1.159	1.159	1.159
**C=O (eq)***	1.162	1.162	1.162	1.162	1.162	1.162
**N=C (azomethine)**	1.309	1.308	1.289	1.307	1.289	1.306
**Bond angle (°)**						
**N-Re-N(L)**	87.61	85.54	89.20	86.04	86.61	86.75
**N-Re-N**	74.38	74.52	74.29	74.41	74.42	74.44
**N-Re-C(eq)**	172.07	171.72	171.88	171.68	171.63	172.02
**C(axial)-Re-N(L)**	179.56	179.56	179.23	179.06	179.72	179.64
**C(eq)-Re-C(axial)**	89.29	89.53	89.01	89.44	89.46	89.43

**average values of two bond distances*

Table S4. Calculated C-O frequencies for **R1** to **R10** complexes.

Complex	ν_{CO} (cm^{-1})		
**R1**	1950	1966	2033
**R2**	1952	1967	2034
**R3**	1951	1965	2032
**R4**	1951	1965	2032
**R5**	1949	1966	2033
**R6**	1952	1967	2034
**R7**	1952	1966	2032
**R8**	1952	1969	2034
**R9**	1952	1965	2032
**R10**	1951	1968	2033

Table S5. Geometrical parameters for the atoms involved in the intramolecular hydrogen bond for **R1** to **R10** complexes in the singlet state.

Complex	Singlet state						
	Bond length (Å)			Bond angle (°)			
	C-O	O-H	H···A*	$C_7N_4=C_{13}C_{14}$	$C_{13}C_{14}C_{15}O$	$C_{14}C_{15}OH$	$C_{15}OH\cdots A$*
**R1**	1.346	1.009	1.685	174.85	-0.12	0.38	3.38
**R2**	1.344	1.007	1.699	173.76	0.30	0.63	2.20
**R3**	1.355	0.978	2.129	176.44	0.33	-179.53	-0.45
**R4**	1.345	1.007	1.701	175.16	-0.05	0.03	3.65
**R5**	1.346	1.007	1.694	173.79	0.31	-0.79	5.16
**R6**	1.344	1.007	1.703	173.30	0.45	0.12	2.83
**R7**	1.348	0.984	2.268	176.41	0.63	-179.35	-1.01
**R8**	1.342	1.008	1.702	173.68	0.16	-1.47	5.83
**R9**	1.355	0.978	2.125	176.37	0.86	-179.22	-1.01
**R10**	1.344	1.008	1.690	175.27	-0.41	-0.97	5.51

A: N_4, except in **R3, **R9** (A: F_{26}) and **R7** (A: Cl_{26})*

Table S6. Geometrical parameters for the atoms involved in the intramolecular hydrogen bond for **R1** to **R10** complexes in T_1 state.

Complex	Triplet state						
	Bond length (Å)			Bond angle (°)			
	C-O	O-H	H···A*	C_7N_4=$C_{13}C_{14}$	$C_{13}C_{14}C_{15}O$	$C_{14}C_{15}OH$	$C_{15}OH$···A*
**R1**	1.326	1.049	1.555	171.23	-1.97	3.15	3.39
**R2**	1.329	1.035	1.601	169.43	-1.86	2.74	3.63
**R3**	1.355	0.980	2.116	152.86	-3.78	176.83	3.77
**R4**	1.328	1.040	1.584	170.64	-2.61	3.69	3.62
**R5**	1.327	1.042	1.578	171.50	-1.60	2.15	4.27
**R6**	1.329	1.032	1.615	169.37	-1.37	2.09	3.88
**R7**	1.343	0.986	2.259	160.68	-4.05	178.66	1.77
**R8**	1.326	1.038	1.592	170.97	-1.74	2.123	4.50
**R9**	1.352	0.979	2.118	157.22	-3.43	177.97	2.40
**R10**	1.327	1.039	1.585	170.61	-1.96	2.24	4.55

A: N_4, except in **R3, **R9** (A: F_{26}) and **R7** (A: Cl_{26})*

Section 2: Supplementary Figures

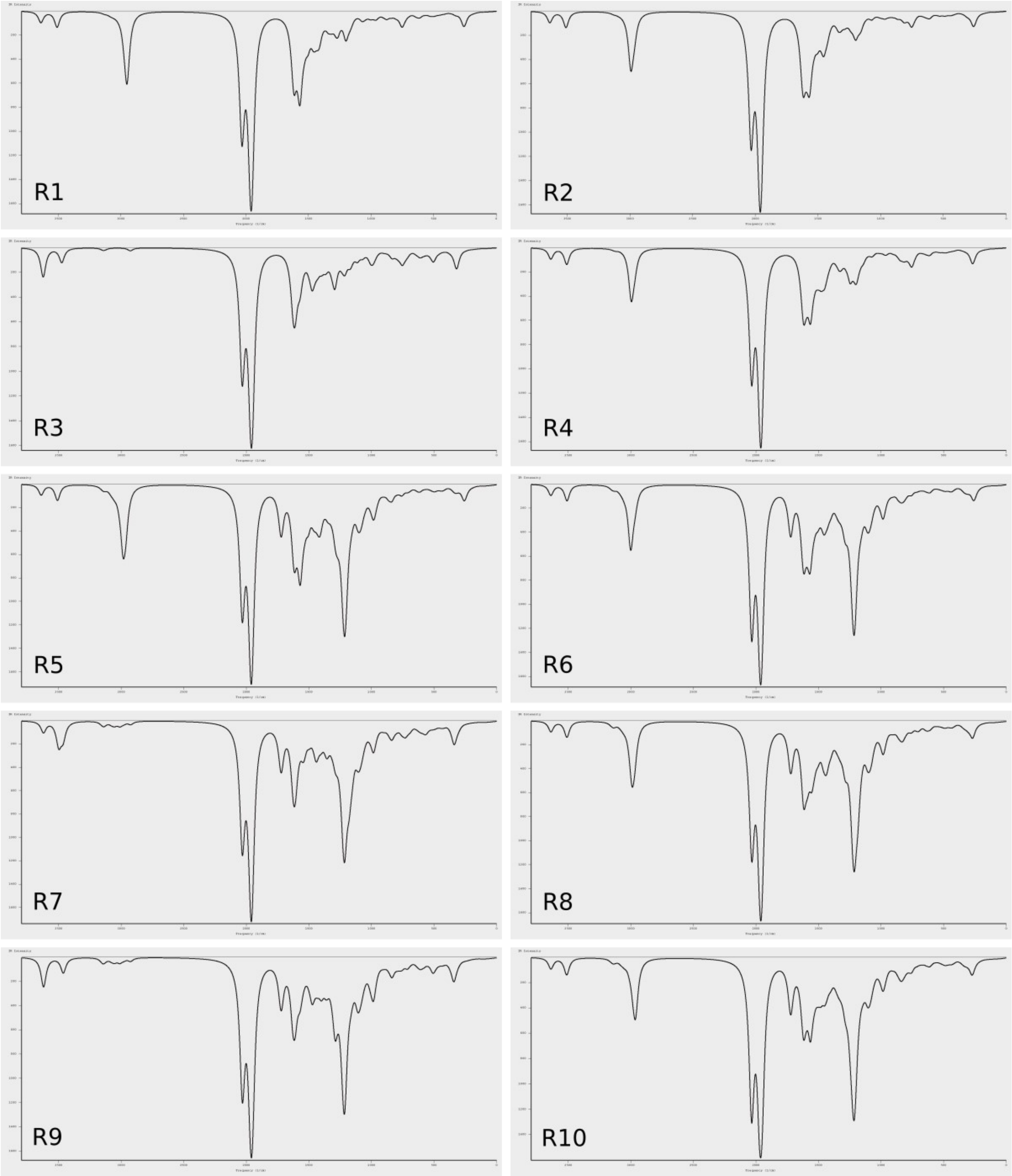

Figure S1. Computed frequencies analysis of **R1** to **R10** complexes.

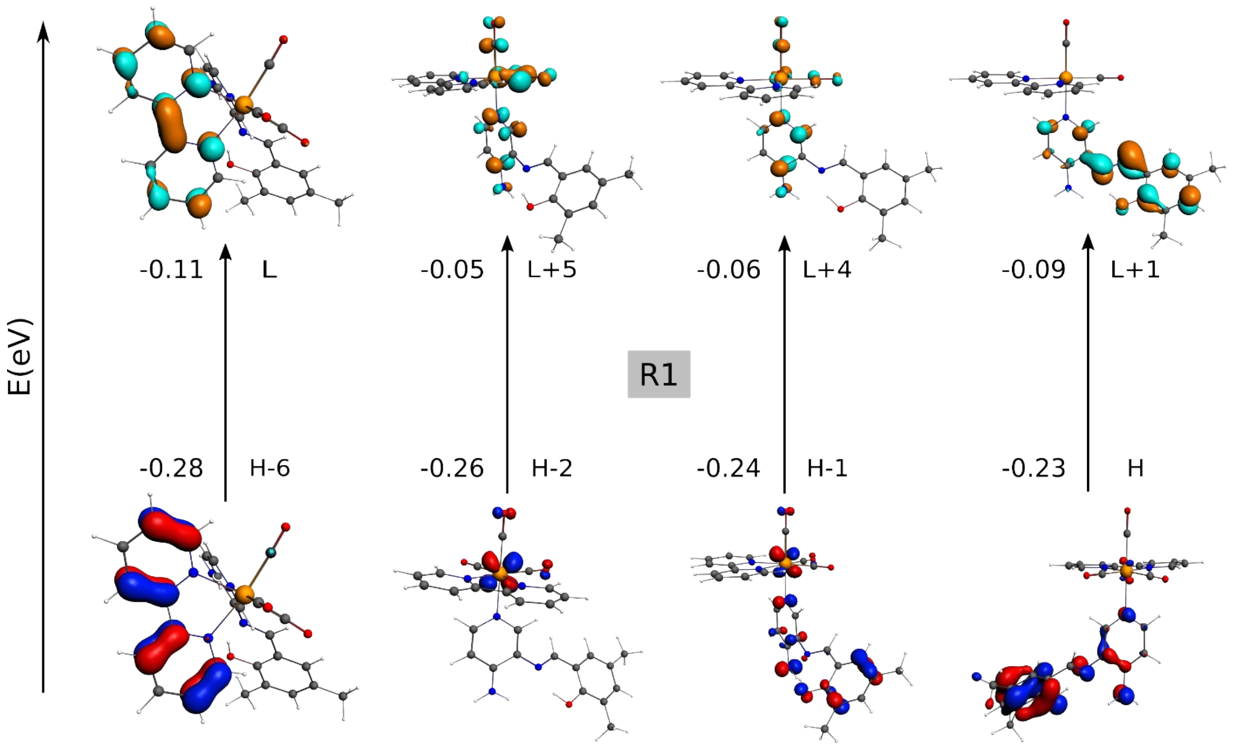

Figure S2. Responsible transitions for the main absorptions for **R1**.

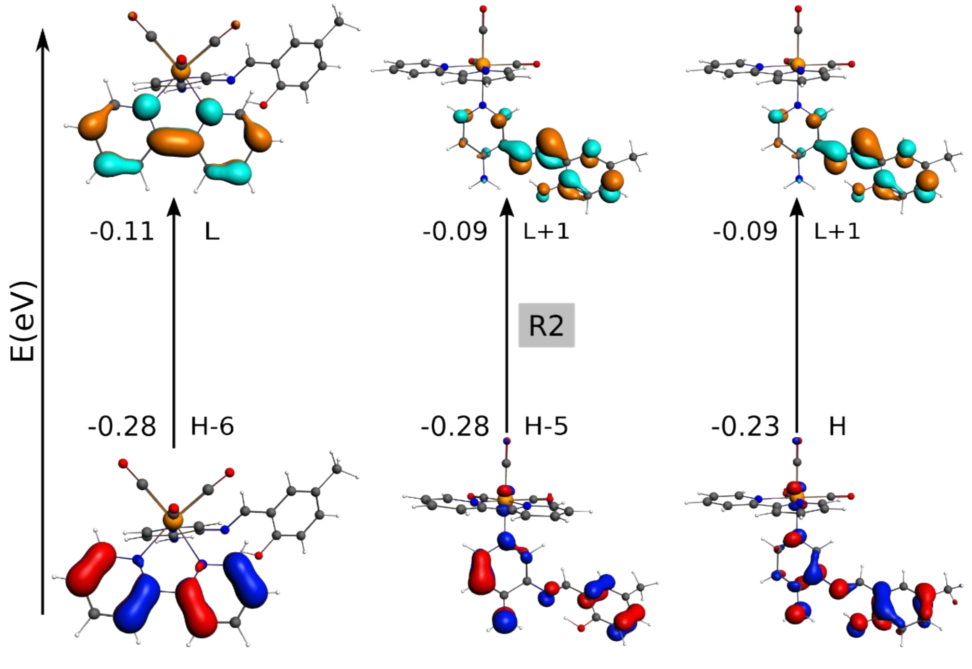

Figure S3. Responsible transitions for the main absorptions for **R2**.

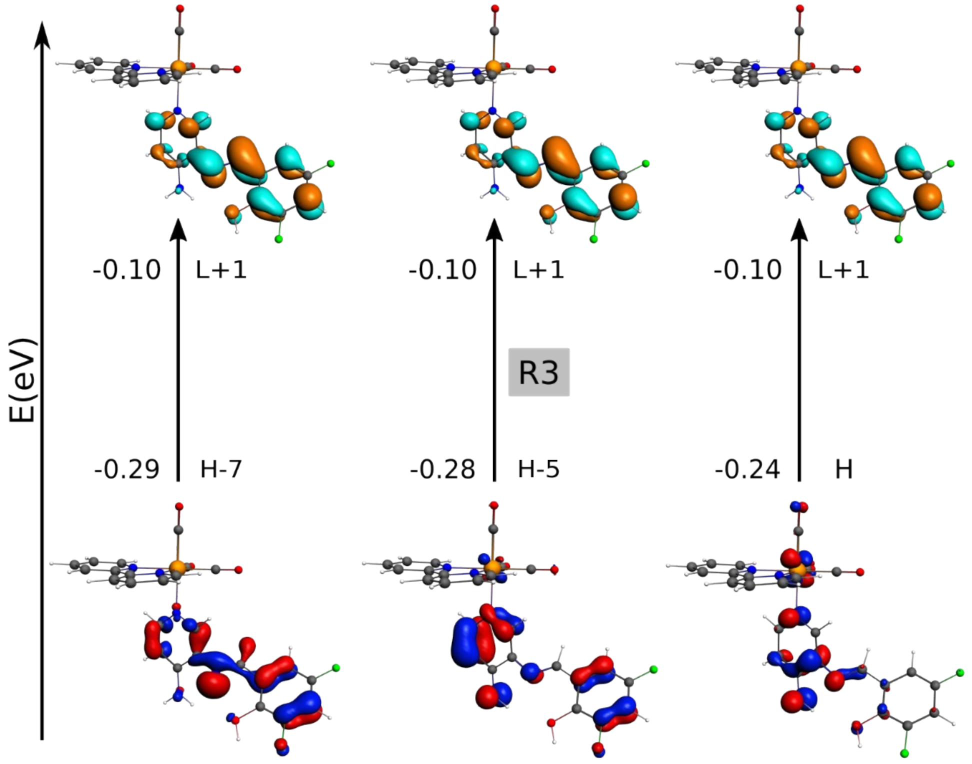

Figure S4. Responsible transitions for the main absorptions for **R3**.

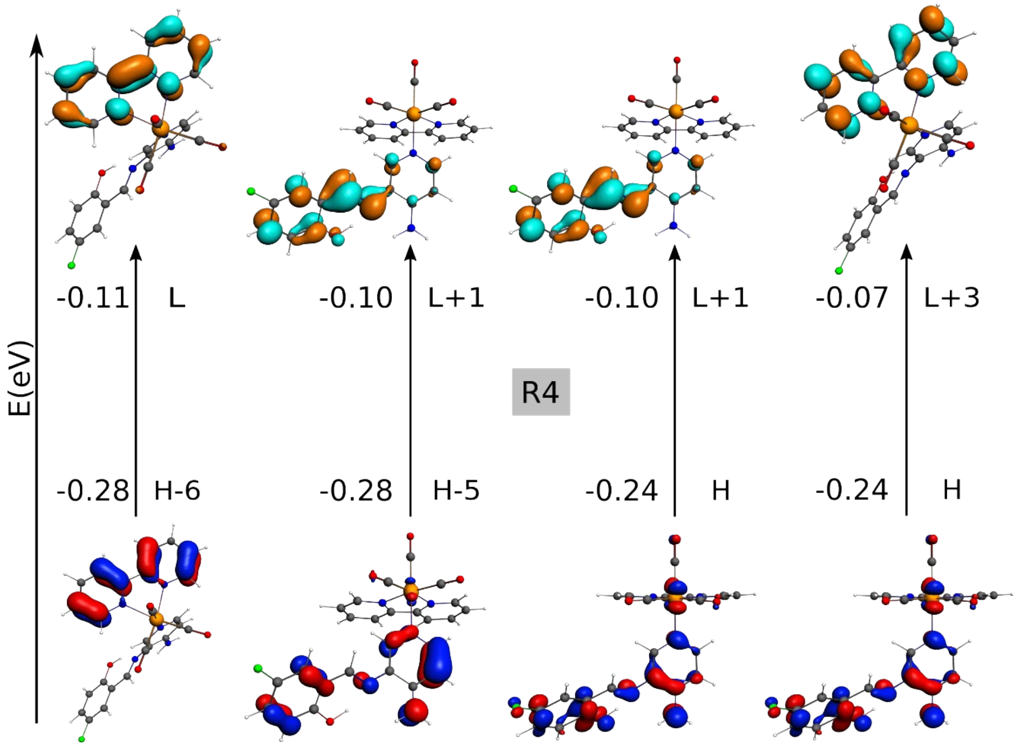

Figure S5. Responsible transitions for the main absorptions for **R4**.

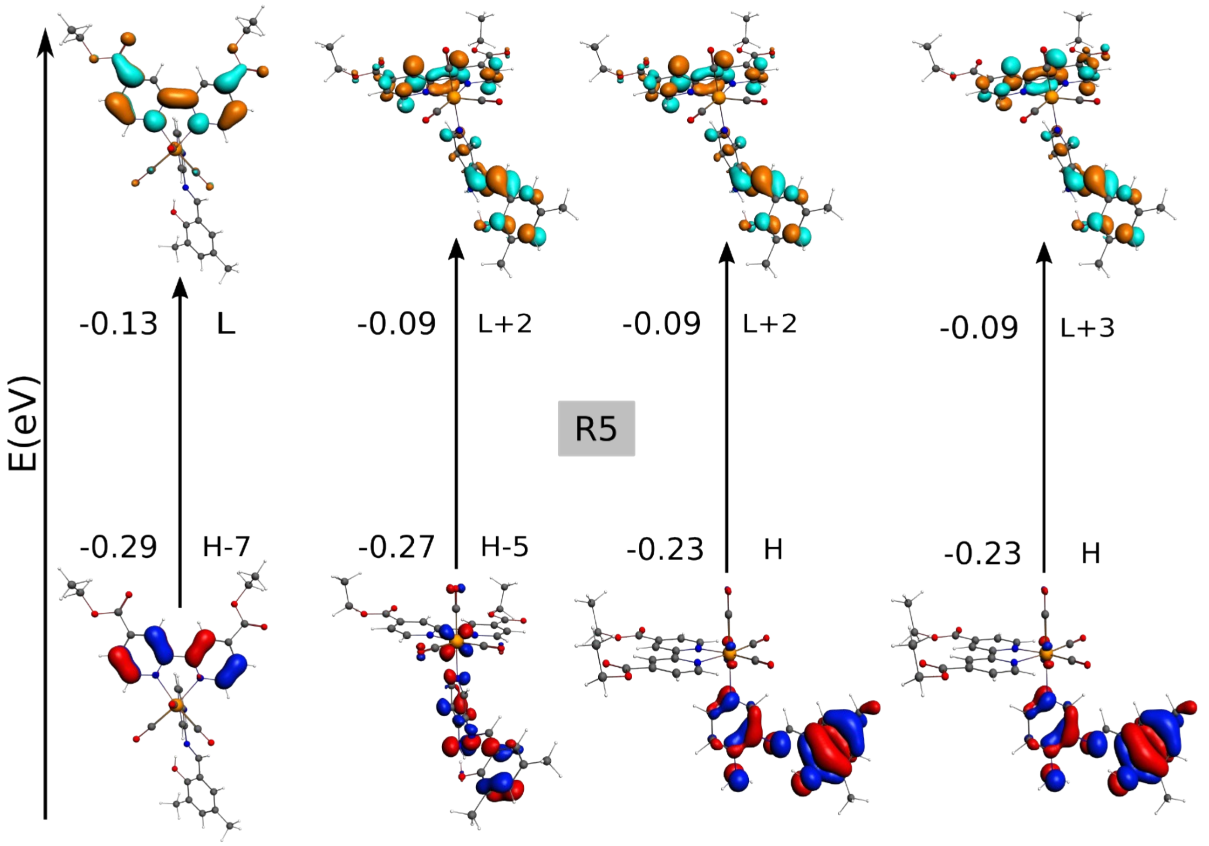

Figure S6. Responsible transitions for the main absorptions for **R5**.

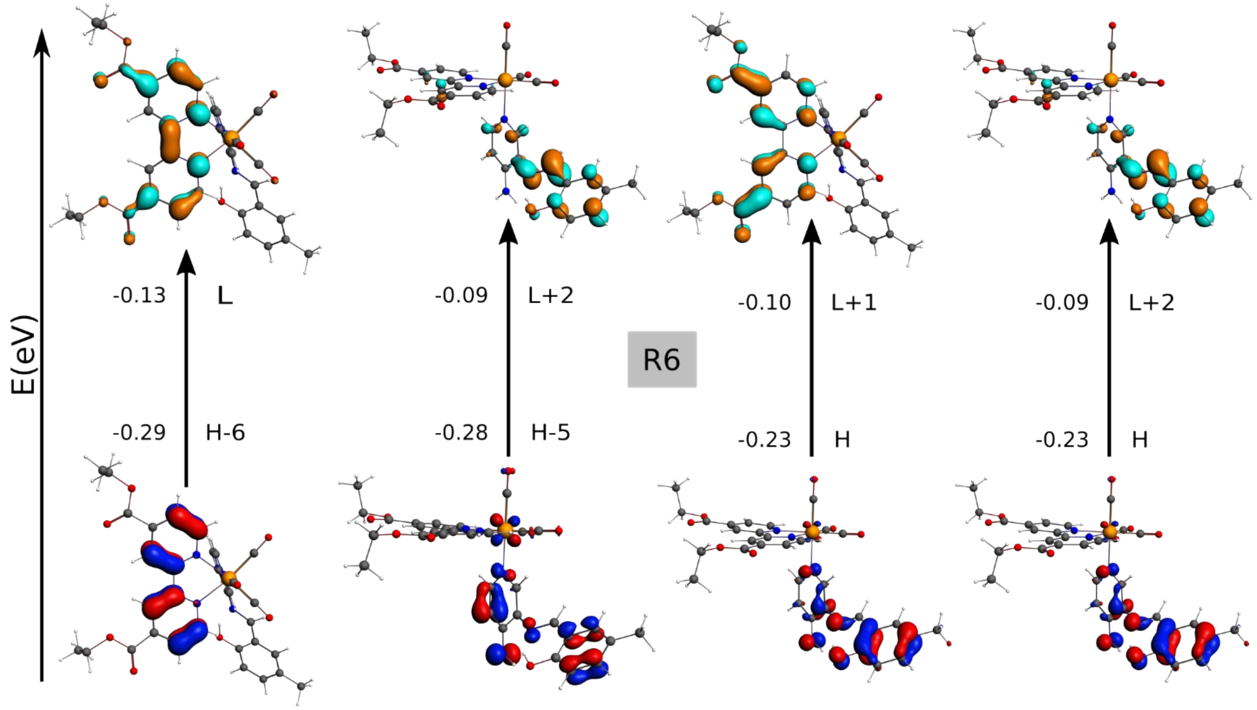

Figure S7. Responsible transitions for the main absorptions for **R6**.

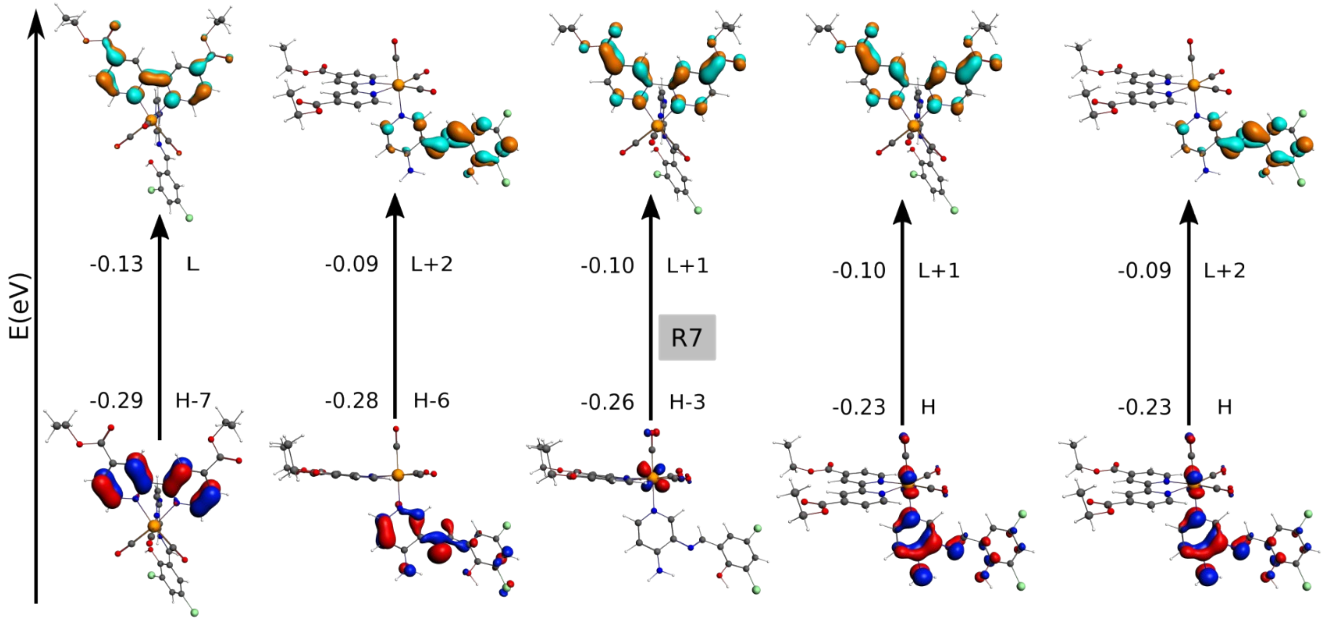

Figure S8. Responsible transitions for the main absorptions for **R7**.

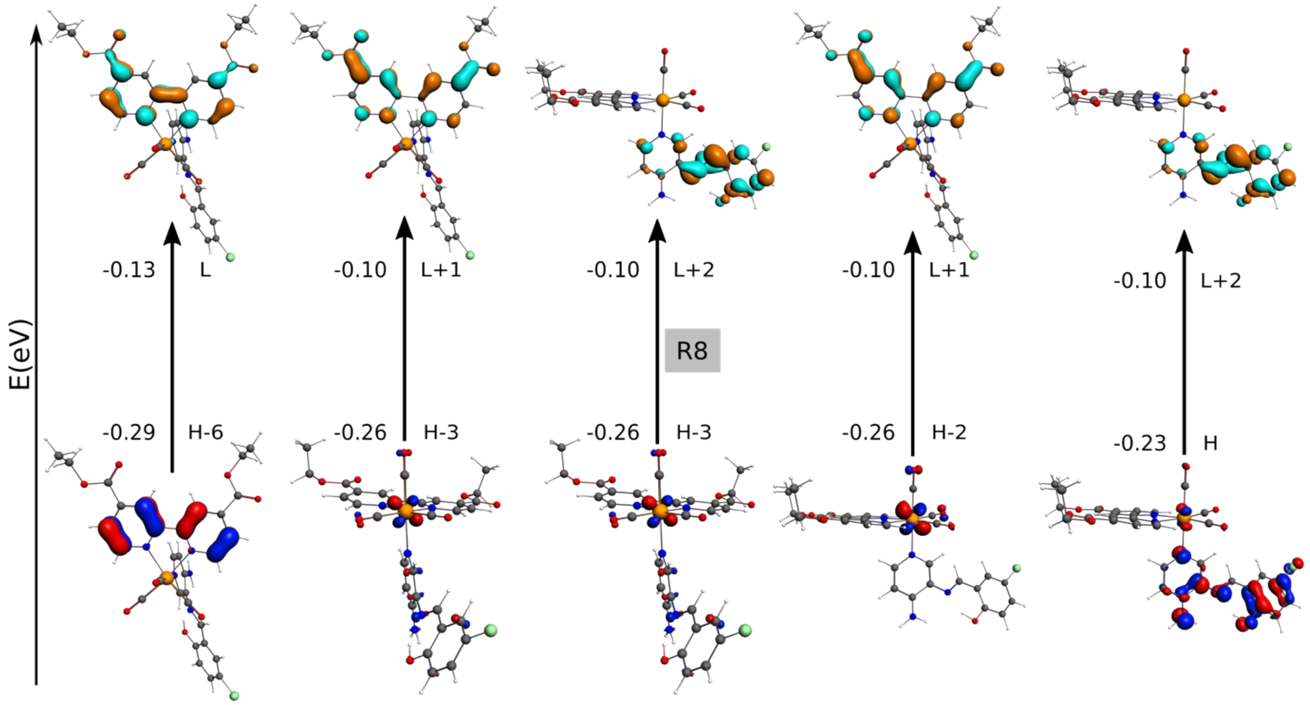

Figure S9. Responsible transitions for the main absorptions for **R8**.

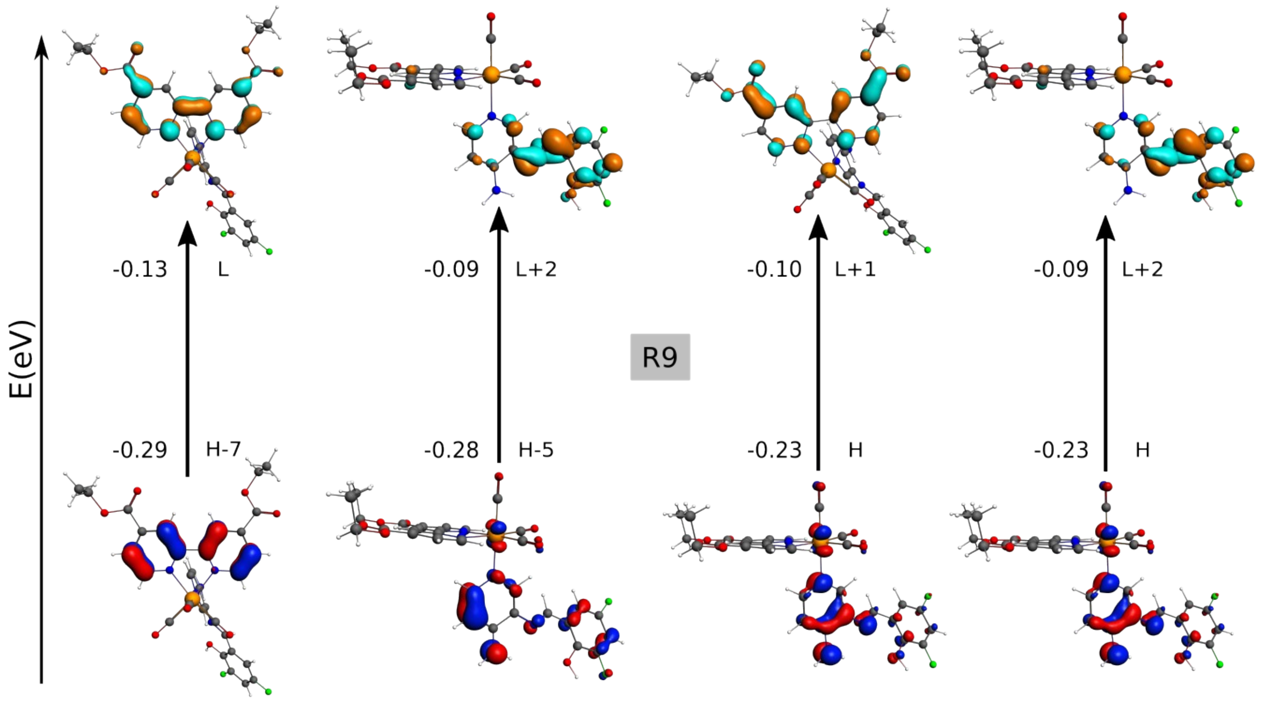

Figure S10. Responsible transitions for the main absorptions for **R9**.

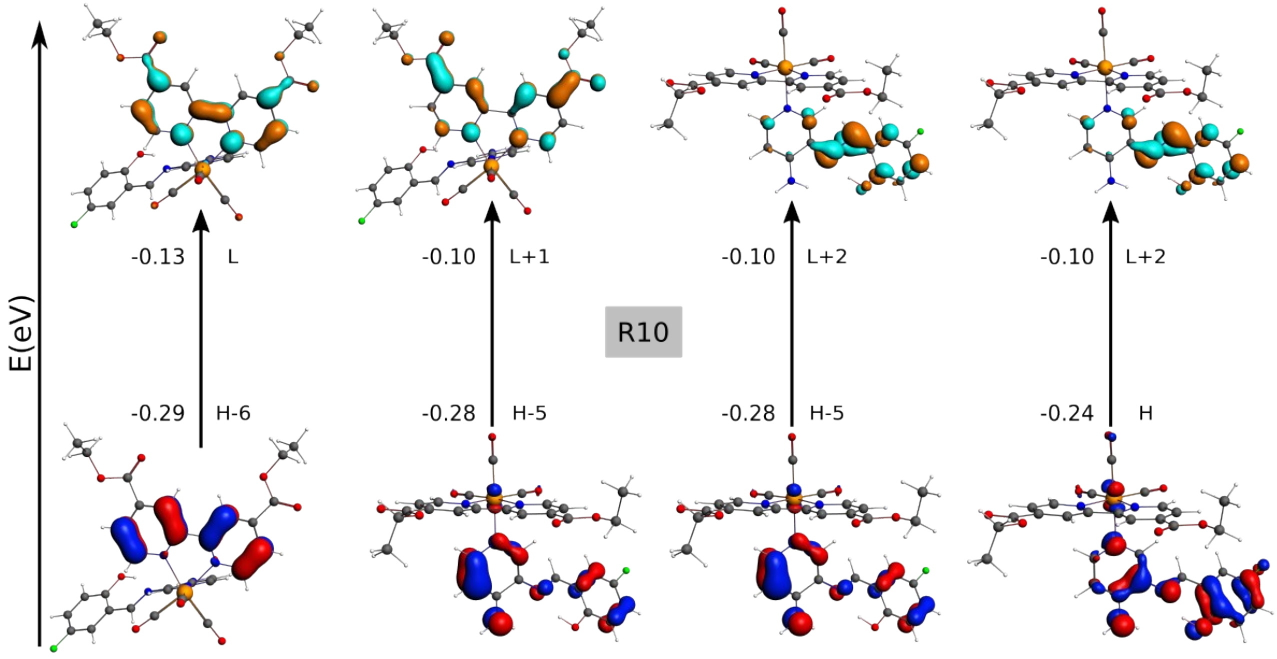

Figure S11. Responsible transitions for the main absorptions for **R10**.

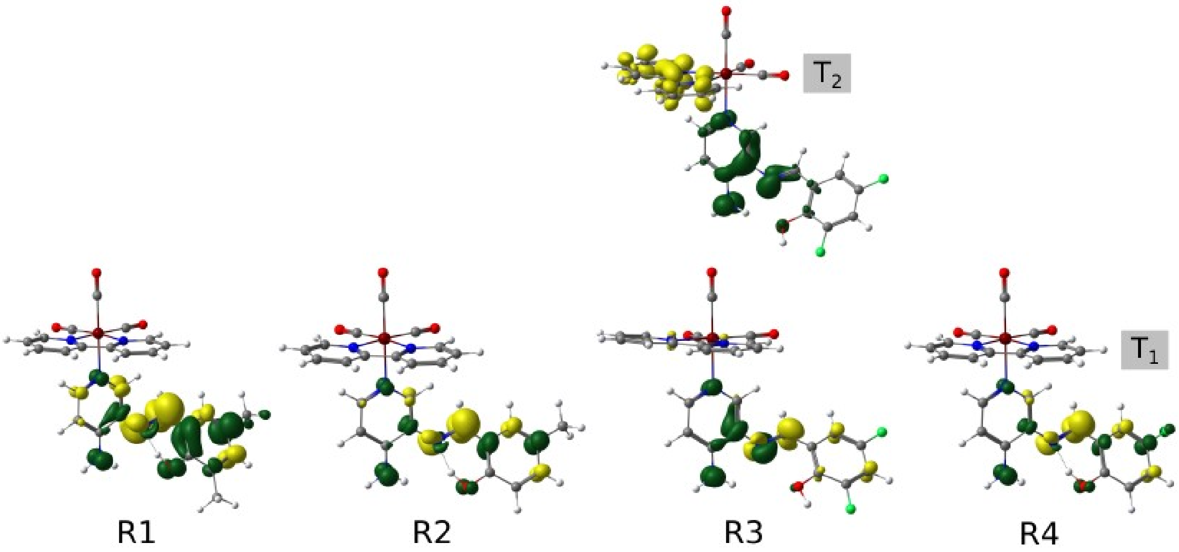

Figure S12. Electron density differences maps between the states involved in the electronic transitions of the emissions for **R1** to **R4** complexes (**yellow** represents a density reduction and **green** density increment as a consequense of the transition).

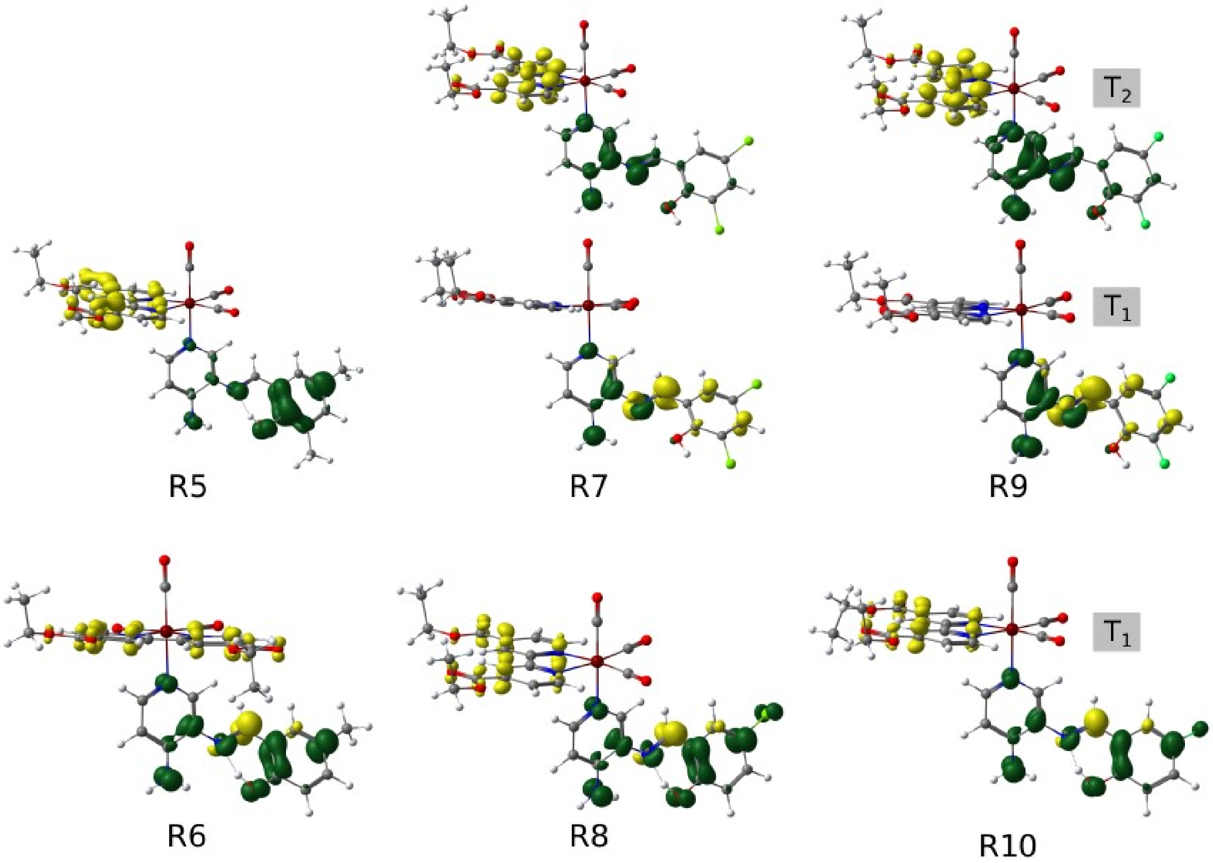

Figure S13. Electron density differences maps between the states involved in the electronic transitions of the emissions for **R5** to R**10** complexes (**yellow** represents a density reduction and **green** density increment as a consequense of the transition).

Section 3. Optimized cartesian coordinates (xyz) of all complexes

Complex R1

Ground state (S_0)

Re	-0.175671000	-0.368119000	1.002179000
O	1.780582000	3.919376000	7.408832000
H	1.178074000	3.331300000	6.852615000
O	2.733899000	-0.910418000	1.897211000
O	-0.994723000	-3.238467000	1.818541000
O	0.589403000	-1.406394000	-1.813385000
N	-0.733224000	0.392122000	3.043914000
N	0.927760000	1.906709000	5.988228000
N	-2.145034000	0.299303000	0.301119000
N	0.073610000	1.742034000	0.453115000
C	0.204431000	0.921607000	3.868744000
H	1.206606000	1.043772000	3.460461000
C	-0.041321000	1.330970000	5.173837000
C	-1.372874000	1.222726000	5.681579000
C	-2.337489000	0.670817000	4.819243000
H	-3.366779000	0.539700000	5.152989000
C	-1.984401000	0.269707000	3.545800000
H	-2.731454000	-0.174660000	2.890832000
C	2.142603000	1.423913000	6.051508000
C	3.189077000	2.055056000	6.795010000
C	2.973115000	3.296444000	7.459134000
C	4.016214000	3.902337000	8.189707000
C	5.240014000	3.244028000	8.239538000
H	6.047826000	3.710020000	8.809792000

C	5.494058000	2.010069000	7.598887000
C	4.460635000	1.435912000	6.880938000
H	4.611493000	0.482133000	6.369915000
C	-3.216852000	-0.514207000	0.174919000
H	-3.067077000	-1.551822000	0.469528000
C	-4.440499000	-0.075211000	-0.309793000
H	-5.267353000	-0.778344000	-0.395992000
C	-4.575603000	1.262019000	-0.679753000
C	-3.476965000	2.104263000	-0.561006000
H	-3.559869000	3.146861000	-0.859735000
C	-2.265062000	1.605966000	-0.071950000
C	-1.037826000	2.411812000	0.031276000
C	-0.974456000	3.766112000	-0.309662000
H	-1.868524000	4.290336000	-0.639698000
C	0.236238000	4.444062000	-0.234837000
C	1.369587000	3.749306000	0.182919000
H	2.343343000	4.231904000	0.249286000
C	1.246184000	2.408160000	0.518268000
H	2.108980000	1.828250000	0.841848000
C	1.632728000	-0.728806000	1.569301000
C	-0.663317000	-2.168130000	1.514551000
C	0.300741000	-1.011953000	-0.762281000
C	6.847354000	1.361949000	7.712168000
H	7.096771000	1.147013000	8.761392000
H	6.887402000	0.417441000	7.155982000
H	7.637595000	2.019410000	7.321744000
C	3.775941000	5.213032000	8.881268000
H	3.475864000	5.990039000	8.163396000

H	2.957014000	5.131786000	9.610454000
H	4.677412000	5.550563000	9.405273000
N	-1.647108000	1.621750000	6.940235000
H	-2.576579000	1.543664000	7.329970000
H	-0.900524000	1.981681000	7.523102000
H	2.392224000	0.481699000	5.535284000
H	-5.521292000	1.641852000	-1.064976000
H	0.294429000	5.498096000	-0.504181000

Complex R1

Triplet state (T_1)

Re	-0.200742000	-0.391609000	0.996752000
O	1.681732000	3.491687000	7.743547000
H	1.155088000	2.884980000	7.068490000
O	2.725298000	-0.919827000	1.844943000
O	-1.021137000	-3.226039000	1.946310000
O	0.476932000	-1.555216000	-1.790943000
N	-0.696453000	0.461483000	3.025061000
N	1.019485000	1.729109000	6.037906000
N	-2.182026000	0.252175000	0.317758000
N	0.054404000	1.688642000	0.376978000
C	0.272736000	0.865158000	3.864737000
H	1.289336000	0.852377000	3.478936000
C	0.046620000	1.318489000	5.184330000
C	-1.322309000	1.372102000	5.637508000
C	-2.321546000	0.973140000	4.735905000
H	-3.370293000	0.990679000	5.031944000
C	-1.980343000	0.520384000	3.475818000

H	-2.750882000	0.177248000	2.790272000
C	2.325448000	1.431705000	5.881344000
C	3.304499000	2.045267000	6.703408000
C	2.941228000	3.083533000	7.662254000
C	3.910644000	3.685567000	8.513678000
C	5.223331000	3.267066000	8.393803000
H	5.983674000	3.717561000	9.033955000
C	5.619775000	2.262763000	7.462877000
C	4.660582000	1.676913000	6.637478000
H	4.961451000	0.901410000	5.930680000
C	-3.261757000	-0.556319000	0.228496000
H	-3.109790000	-1.589718000	0.537428000
C	-4.494814000	-0.121650000	-0.230044000
H	-5.325926000	-0.822137000	-0.286024000
C	-4.632110000	1.217611000	-0.616857000
C	-3.531610000	2.054530000	-0.540480000
H	-3.618854000	3.092828000	-0.853116000
C	-2.300035000	1.562262000	-0.075692000
C	-1.076410000	2.353194000	-0.030776000
C	-1.006784000	3.702621000	-0.414388000
H	-1.907219000	4.224335000	-0.731217000
C	0.206900000	4.368986000	-0.402222000
C	1.356567000	3.675515000	-0.003746000
H	2.335689000	4.150734000	0.009847000
C	1.233048000	2.349529000	0.376700000
H	2.102375000	1.771976000	0.687520000
C	1.616979000	-0.740899000	1.532108000
C	-0.693639000	-2.169204000	1.593305000

C	0.220747000	-1.113307000	-0.750736000
C	7.054722000	1.849938000	7.400206000
H	7.387267000	1.457644000	8.374354000
H	7.231893000	1.082849000	6.638302000
H	7.699233000	2.716286000	7.183769000
C	3.480467000	4.745346000	9.480635000
H	3.043221000	5.605795000	8.953363000
H	2.700152000	4.368942000	10.157751000
H	4.328157000	5.095605000	10.079660000
N	-1.594395000	1.777981000	6.892223000
H	-2.541554000	1.778888000	7.248816000
H	-0.834083000	1.942534000	7.541364000
H	2.654611000	0.673475000	5.161887000
H	-5.585929000	1.596044000	-0.983031000
H	0.263678000	5.413587000	-0.706549000

Complex R2

Ground state (S_0)

Re	-0.039904000	0.052937000	0.110447000
O	6.612534000	-3.011497000	-2.858616000
H	6.097787000	-2.391422000	-2.254327000
O	-0.150356000	-3.026245000	0.374174000
O	-0.125375000	0.360428000	3.188658000
O	-3.135808000	0.097618000	0.045761000
N	2.206139000	0.023073000	0.132056000
N	4.957542000	-2.173886000	-1.013957000
N	0.089313000	2.186690000	-0.380895000
N	0.163602000	0.148009000	-2.071184000

C	2.891613000	-1.016860000	-0.403889000
H	2.304130000	-1.796136000	-0.887582000
C	4.276514000	-1.127046000	-0.400540000
C	5.041454000	-0.071042000	0.184242000
C	4.320359000	0.996590000	0.749688000
H	4.843146000	1.821330000	1.233938000
C	2.940285000	1.002676000	0.709741000
H	2.386748000	1.824682000	1.160367000
C	4.573550000	-3.415934000	-0.874639000
C	5.176117000	-4.495150000	-1.597371000
C	6.183801000	-4.254257000	-2.577522000
C	6.738327000	-5.340348000	-3.267166000
C	6.314253000	-6.628930000	-2.985395000
H	6.764545000	-7.460492000	-3.531611000
C	5.322317000	-6.905747000	-2.016696000
C	4.769790000	-5.828372000	-1.343871000
H	3.998988000	-5.997906000	-0.587984000
C	-0.047726000	3.180662000	0.524004000
H	-0.225928000	2.869690000	1.552278000
C	0.020175000	4.523592000	0.181838000
H	-0.105380000	5.281305000	0.953773000
C	0.243968000	4.866555000	-1.150545000
C	0.376823000	3.851587000	-2.089870000
H	0.535966000	4.101030000	-3.136491000
C	0.289655000	2.514459000	-1.689849000
C	0.351772000	1.380491000	-2.625532000
C	0.547319000	1.527038000	-4.002200000
H	0.702581000	2.514317000	-4.431459000

C	0.537747000	0.408949000	-4.826801000
C	0.324653000	-0.844266000	-4.255655000
H	0.295169000	-1.749627000	-4.859881000
C	0.144274000	-0.931348000	-2.882272000
H	-0.033303000	-1.890512000	-2.398516000
C	-0.111424000	-1.866241000	0.301528000
C	-0.099353000	0.220007000	2.036448000
C	-1.977129000	0.085123000	0.065168000
C	4.904504000	-8.324038000	-1.738655000
H	5.746490000	-8.914781000	-1.348951000
H	4.093627000	-8.367155000	-1.001502000
H	4.558419000	-8.823632000	-2.654926000
N	6.387744000	-0.140808000	0.192769000
H	6.953305000	0.587816000	0.606476000
H	6.848648000	-0.948337000	-0.209977000
H	3.764228000	-3.671723000	-0.169983000
H	0.304554000	5.910609000	-1.456085000
H	0.687558000	0.515791000	-5.900680000
H	7.505723000	-5.146782000	-4.016480000

Complex R2

Triplet state (T_1)

Re	-0.001655000	0.100336000	0.095797000
O	7.045284000	-3.152304000	-1.946349000
H	6.366779000	-2.554760000	-1.442788000
O	-0.102817000	-2.973411000	0.416744000
O	0.001737000	0.476359000	3.173005000
O	-3.097463000	0.153509000	0.134469000

N	2.246427000	0.049764000	0.069926000
N	4.998308000	-2.298007000	-0.653467000
N	0.111586000	2.223359000	-0.434450000
N	0.135896000	0.141379000	-2.084213000
C	2.911849000	-1.046175000	-0.328819000
H	2.315687000	-1.851372000	-0.751864000
C	4.317561000	-1.199306000	-0.251128000
C	5.071719000	-0.081847000	0.271886000
C	4.357431000	1.066978000	0.651129000
H	4.881280000	1.934782000	1.051403000
C	2.980242000	1.091685000	0.553191000
H	2.421700000	1.966533000	0.877735000
C	4.411824000	-3.497456000	-0.867528000
C	5.122377000	-4.547575000	-1.496023000
C	6.469700000	-4.348342000	-2.019514000
C	7.170111000	-5.413098000	-2.616280000
C	6.574470000	-6.650693000	-2.732387000
H	7.116332000	-7.471686000	-3.202834000
C	5.251011000	-6.882636000	-2.241517000
C	4.560188000	-5.835676000	-1.641798000
H	3.553876000	-6.010178000	-1.255869000
C	0.004014000	3.240634000	0.448570000
H	-0.143643000	2.954796000	1.489237000
C	0.065046000	4.574056000	0.076595000
H	-0.036317000	5.349448000	0.833795000
C	0.248827000	4.884759000	-1.275970000
C	0.349760000	3.852003000	-2.193333000
H	0.477430000	4.077947000	-3.249667000

C	0.271690000	2.516770000	-1.765401000
C	0.292828000	1.373178000	-2.670257000
C	0.417209000	1.486039000	-4.064233000
H	0.545519000	2.464878000	-4.520982000
C	0.364418000	0.355972000	-4.862703000
C	0.179534000	-0.892791000	-4.256524000
H	0.114612000	-1.808507000	-4.841395000
C	0.073497000	-0.951932000	-2.877035000
H	-0.079609000	-1.901668000	-2.366587000
C	-0.070394000	-1.813063000	0.321606000
C	-0.001926000	0.312721000	2.023356000
C	-1.939285000	0.138543000	0.114494000
C	4.648768000	-8.244336000	-2.375367000
H	5.274842000	-8.996857000	-1.870604000
H	3.638432000	-8.293411000	-1.954363000
H	4.602389000	-8.545363000	-3.433903000
N	6.407327000	-0.179470000	0.395484000
H	6.954926000	0.572916000	0.793732000
H	6.864868000	-1.064069000	0.206558000
H	3.396449000	-3.702695000	-0.509760000
H	0.302096000	5.921370000	-1.607177000
H	0.454596000	0.442615000	-5.944987000
H	8.176725000	-5.222112000	-2.987839000

Complex R3

Ground state (S_0)

Re	-0.030891000	-0.306923000	0.884438000
O	1.946887000	3.840371000	7.734317000
H	2.107161000	4.581603000	8.351694000
O	2.909742000	-0.658828000	1.772970000
O	-0.654787000	-3.230346000	1.686467000
O	0.807923000	-1.282889000	-1.932310000
N	-0.636165000	0.411304000	2.928871000
N	0.945470000	1.915925000	5.929364000
N	-2.036338000	0.223400000	0.170700000
N	0.071162000	1.820733000	0.360440000
C	0.283275000	0.949596000	3.769476000
H	1.283780000	1.104169000	3.368648000
C	0.016375000	1.332573000	5.077752000
C	-1.321671000	1.204535000	5.566085000
C	-2.265450000	0.633534000	4.692802000
H	-3.296416000	0.481475000	5.011949000
C	-1.887952000	0.250219000	3.420241000
H	-2.616876000	-0.206759000	2.753397000
C	2.160056000	1.483255000	5.954723000
C	3.241674000	2.048989000	6.740455000
C	3.120492000	3.179302000	7.583597000
C	4.262298000	3.607531000	8.274430000
C	5.493507000	2.988391000	8.185573000
H	6.351310000	3.356777000	8.746064000
C	5.584658000	1.874675000	7.350780000

C	4.497087000	1.406411000	6.641332000
H	4.611021000	0.532045000	6.001106000
C	-3.041022000	-0.666534000	0.011139000
H	-2.821654000	-1.692634000	0.302307000
C	-4.281379000	-0.315849000	-0.502070000
H	-5.050440000	-1.078339000	-0.615512000
C	-4.506413000	1.010617000	-0.866613000
C	-3.478110000	1.931968000	-0.710228000
H	-3.630621000	2.967593000	-1.005668000
C	-2.245537000	1.521624000	-0.192343000
C	-1.085739000	2.416306000	-0.050136000
C	-1.125912000	3.782738000	-0.342367000
H	-2.056852000	4.248184000	-0.658289000
C	0.027497000	4.549559000	-0.232418000
C	1.209716000	3.929426000	0.166747000
H	2.143224000	4.482771000	0.256004000
C	1.188947000	2.571958000	0.454560000
H	2.092231000	2.049023000	0.764314000
C	1.797815000	-0.546932000	1.449125000
C	-0.397253000	-2.138484000	1.388149000
C	0.490432000	-0.911863000	-0.880965000
N	-1.594744000	1.599471000	6.824412000
H	-2.538441000	1.599822000	7.187355000
H	-0.860224000	2.079984000	7.337411000
H	2.454365000	0.597753000	5.360547000
H	-5.466578000	1.321601000	-1.277074000
H	0.004386000	5.614412000	-0.461130000
F	6.774291000	1.244137000	7.240689000

F	4.107861000	4.700671000	9.070365000

Complex R3

Triplet state (T_1)

Re	0.004823000	-0.185983000	0.920871000
O	1.943253000	3.559323000	7.715527000
H	2.034797000	4.294054000	8.356389000
O	2.994170000	-0.360946000	1.688261000
O	-0.445673000	-3.088358000	1.908056000
O	0.781461000	-1.279154000	-1.867336000
N	-0.561081000	0.620313000	2.961446000
N	0.980221000	1.446779000	6.194674000
N	-2.039081000	0.199655000	0.218031000
N	0.002209000	1.902816000	0.265915000
C	0.374003000	0.884593000	3.881952000
H	1.415375000	0.784621000	3.583481000
C	0.087090000	1.251885000	5.231369000
C	-1.315337000	1.435123000	5.580945000
C	-2.274315000	1.174007000	4.590490000
H	-3.337888000	1.279567000	4.803239000
C	-1.871646000	0.766895000	3.332452000
H	-2.612868000	0.537950000	2.572047000
C	2.287649000	1.120364000	6.116726000
C	3.324379000	1.764539000	6.829496000
C	3.144978000	2.937594000	7.641628000
C	4.238053000	3.444744000	8.352721000
C	5.507435000	2.907897000	8.296230000
H	6.336612000	3.334716000	8.856500000

C	5.671346000	1.781705000	7.469095000
C	4.639664000	1.219921000	6.756220000
H	4.828037000	0.336125000	6.148222000
C	-3.009574000	-0.738873000	0.144593000
H	-2.748629000	-1.725082000	0.525520000
C	-4.265307000	-0.484845000	-0.384655000
H	-5.004134000	-1.283382000	-0.423581000
C	-4.543252000	0.798678000	-0.865575000
C	-3.555707000	1.769081000	-0.798468000
H	-3.751994000	2.767701000	-1.182247000
C	-2.299500000	1.459095000	-0.255630000
C	-1.185451000	2.405542000	-0.196308000
C	-1.281347000	3.742361000	-0.609115000
H	-2.229750000	4.135067000	-0.968882000
C	-0.166391000	4.565042000	-0.564216000
C	1.043826000	4.037958000	-0.104788000
H	1.949695000	4.639997000	-0.061189000
C	1.082506000	2.712356000	0.298977000
H	2.006392000	2.261600000	0.657339000
C	1.862697000	-0.311069000	1.416587000
C	-0.258617000	-2.003193000	1.540741000
C	0.487712000	-0.865002000	-0.825956000
N	-1.592783000	1.840486000	6.824100000
H	-2.540857000	1.966355000	7.155112000
H	-0.802373000	1.998869000	7.448198000
H	2.577611000	0.229500000	5.542867000
H	-5.516472000	1.033849000	-1.295246000
H	-0.235681000	5.602998000	-0.888182000

F	6.902100000	1.236382000	7.396013000
F	3.994795000	4.547829000	9.113020000

Complex R4

Ground state (S_0)

Re	0.031751000	-0.212633000	0.656046000
O	1.454720000	3.467380000	7.595243000
H	0.937905000	2.873483000	6.967231000
O	2.955210000	-0.752214000	1.507804000
O	-0.652291000	-3.215586000	0.953554000
O	0.846019000	-0.724239000	-2.287344000
N	-0.567087000	0.170188000	2.790956000
N	0.908842000	1.554564000	5.893207000
N	-1.971686000	0.477073000	0.090104000
N	0.190990000	1.967170000	0.457448000
C	0.292676000	0.741919000	3.668622000
H	1.243646000	1.091110000	3.268111000
C	0.023511000	0.927708000	5.019303000
C	-1.249075000	0.520099000	5.524396000
C	-2.133674000	-0.073405000	4.604802000
H	-3.112386000	-0.426812000	4.928942000
C	-1.760928000	-0.234048000	3.285695000
H	-2.441913000	-0.712149000	2.583864000
C	2.186311000	1.286875000	5.868807000
C	3.136470000	1.986752000	6.683503000
C	2.735866000	3.064741000	7.529067000
C	3.694789000	3.722758000	8.313224000
C	5.022973000	3.326649000	8.271914000

H	5.777542000	3.826529000	8.879094000
C	5.406229000	2.266033000	7.441776000
C	4.496554000	1.598515000	6.653716000
H	4.824163000	0.774702000	6.018876000
C	-3.016995000	-0.345743000	-0.146319000
H	-2.817868000	-1.412141000	-0.051068000
C	-4.275007000	0.120015000	-0.500881000
H	-5.078944000	-0.591250000	-0.683694000
C	-4.474009000	1.494836000	-0.616618000
C	-3.401965000	2.348457000	-0.387876000
H	-3.535727000	3.423317000	-0.487386000
C	-2.153488000	1.823197000	-0.040427000
C	-0.953484000	2.650375000	0.165236000
C	-0.946038000	4.042486000	0.036024000
H	-1.864654000	4.575858000	-0.197989000
C	0.240283000	4.747700000	0.197125000
C	1.407270000	4.041743000	0.482621000
H	2.364123000	4.546352000	0.606695000
C	1.340615000	2.660811000	0.604372000
H	2.231641000	2.071533000	0.815188000
C	1.851686000	-0.576046000	1.186225000
C	-0.370878000	-2.094427000	0.845828000
C	0.538092000	-0.529650000	-1.187195000
N	-1.545947000	0.688498000	6.828495000
H	-2.435165000	0.395666000	7.210294000
H	-0.857659000	1.092965000	7.452103000
H	2.576553000	0.484982000	5.220068000
H	-5.448836000	1.897239000	-0.890372000

H	0.254402000	5.832184000	0.093456000
H	3.372922000	4.543751000	8.953017000
F	6.708033000	1.894730000	7.420127000

Complex R4

Triplet state (T_1)

Re	0.014003000	-0.202908000	0.692417000
O	1.458338000	2.955023000	7.923214000
H	0.997191000	2.392226000	7.180711000
O	2.940538000	-0.698329000	1.557922000
O	-0.683339000	-3.181232000	1.190781000
O	0.819144000	-0.910802000	-2.212896000
N	-0.569294000	0.310938000	2.804297000
N	0.993416000	1.365116000	5.974376000
N	-1.984700000	0.445238000	0.068600000
N	0.175688000	1.958271000	0.391595000
C	0.345535000	0.725977000	3.696077000
H	1.348950000	0.906388000	3.317918000
C	0.076982000	0.955995000	5.067206000
C	-1.283409000	0.753538000	5.513428000
C	-2.228760000	0.346931000	4.557993000
H	-3.265166000	0.173533000	4.846961000
C	-1.843677000	0.125927000	3.250019000
H	-2.566810000	-0.224518000	2.517835000
C	2.329105000	1.289937000	5.763188000
C	3.232026000	1.909338000	6.657010000
C	2.759111000	2.734434000	7.769636000
C	3.662818000	3.316217000	8.680515000

C	5.023169000	3.141470000	8.521681000
H	5.749467000	3.583058000	9.201571000
C	5.473981000	2.357877000	7.432253000
C	4.628211000	1.753095000	6.521701000
H	5.048018000	1.149025000	5.717695000
C	-3.022724000	-0.389250000	-0.160492000
H	-2.821477000	-1.450713000	-0.022097000
C	-4.275451000	0.055749000	-0.553286000
H	-5.072095000	-0.665202000	-0.728015000
C	-4.477377000	1.429708000	-0.719430000
C	-3.416977000	2.295018000	-0.500703000
H	-3.554047000	3.364936000	-0.641360000
C	-2.166375000	1.791723000	-0.111400000
C	-0.979689000	2.625156000	0.075374000
C	-0.975585000	4.017915000	-0.090759000
H	-1.897668000	4.539708000	-0.337344000
C	0.204196000	4.731846000	0.048314000
C	1.381400000	4.039527000	0.348896000
H	2.335590000	4.552777000	0.453801000
C	1.321481000	2.664744000	0.512504000
H	2.215886000	2.087038000	0.740725000
C	1.834424000	-0.535987000	1.232545000
C	-0.399416000	-2.070692000	1.007271000
C	0.514193000	-0.641047000	-1.128302000
N	-1.592205000	0.936324000	6.809492000
H	-2.526062000	0.759557000	7.157563000
H	-0.853584000	1.120325000	7.478774000
H	2.740684000	0.694169000	4.941362000

H	-5.448634000	1.817194000	-1.025577000
H	0.211658000	5.813147000	-0.084694000
H	3.256886000	3.913088000	9.496528000
F	6.798513000	2.192078000	7.291614000

Complex R5

Ground state (S_0)

Re	0.407968000	1.886522000	1.198802000
O	7.271387000	2.175985000	-2.769326000
H	6.681812000	2.305395000	-1.962442000
O	0.163333000	3.165090000	-1.609773000
O	0.970791000	-0.891192000	-0.039191000
O	-2.614835000	1.214361000	1.108991000
N	2.603581000	2.355638000	1.257374000
N	5.586204000	1.675655000	-0.834046000
N	0.573048000	1.288515000	3.298303000
N	-0.014695000	3.696767000	2.363450000
C	3.414036000	1.896792000	0.267936000
H	2.932412000	1.354626000	-0.543861000
C	4.784612000	2.115407000	0.213010000
C	5.388039000	2.907198000	1.239759000
C	4.540252000	3.382232000	2.256385000
H	4.939898000	3.983909000	3.072594000
C	3.191002000	3.085676000	2.232942000
H	2.544246000	3.457233000	3.025437000
C	5.411674000	0.498131000	-1.378625000
C	6.127063000	0.061683000	-2.537137000
C	7.042680000	0.923548000	-3.206570000

C	7.731328000	0.475870000	-4.353363000
C	7.486699000	-0.821116000	-4.791458000
H	8.020506000	-1.169175000	-5.679703000
C	6.588565000	-1.708354000	-4.155253000
C	5.920246000	-1.248983000	-3.034942000
H	5.214061000	-1.899106000	-2.513059000
C	0.920892000	0.048028000	3.706206000
H	1.168859000	-0.663402000	2.920920000
C	0.950750000	-0.319728000	5.040610000
H	1.226944000	-1.330197000	5.338874000
C	0.608146000	0.622536000	6.013576000
C	0.260073000	1.907716000	5.602559000
H	-0.004612000	2.652916000	6.348687000
C	0.250309000	2.221006000	4.241429000
C	-0.105959000	3.550588000	3.719786000
C	-0.527977000	4.604459000	4.528094000
H	-0.627713000	4.498417000	5.606940000
C	-0.857758000	5.832645000	3.959221000
C	-0.735591000	5.981935000	2.575221000
H	-0.977268000	6.924546000	2.088732000
C	-0.313331000	4.897402000	1.820414000
H	-0.220167000	4.970055000	0.738321000
C	0.259689000	2.664989000	-0.566522000
C	0.762815000	0.162596000	0.406024000
C	-1.484525000	1.467035000	1.146500000
C	6.382424000	-3.097086000	-4.696887000
H	7.325404000	-3.662880000	-4.708664000
H	5.658971000	-3.659166000	-4.093760000

H	6.010905000	-3.068779000	-5.731627000
C	8.687545000	1.396620000	-5.053883000
H	8.180593000	2.314394000	-5.385071000
H	9.497438000	1.714417000	-4.381622000
H	9.131924000	0.907658000	-5.928394000
N	6.714104000	3.148711000	1.209809000
H	7.160874000	3.738651000	1.898250000
H	7.268079000	2.784331000	0.443452000
H	4.696283000	-0.216044000	-0.937210000
C	0.621765000	0.188518000	7.455382000
O	0.925207000	-0.938710000	7.789366000
O	0.263209000	1.189964000	8.277936000
C	-1.350939000	6.916983000	4.880091000
O	-1.457839000	6.750901000	6.079846000
O	-1.645470000	8.038427000	4.204584000
C	-2.198548000	9.149312000	4.992658000
H	-1.743733000	9.119651000	5.989512000
H	-1.861806000	10.041766000	4.453671000
C	0.200172000	0.859459000	9.709322000
H	0.994263000	0.135128000	9.925360000
H	0.418372000	1.812237000	10.204276000
C	-3.708814000	9.058593000	5.055123000
H	-4.147584000	9.050571000	4.049384000
H	-4.030096000	8.160749000	5.597325000
H	-4.099045000	9.934954000	5.590251000
C	-1.167836000	0.325653000	10.078702000
H	-1.956974000	1.043597000	9.821737000
H	-1.369122000	-0.629073000	9.577762000

H	-1.204239000	0.153114000	11.162943000

Complex R5

Triplet state (T_1)

Re	0.397588000	1.805044000	1.273992000
O	7.450635000	1.946825000	-2.420241000
H	6.766794000	2.009991000	-1.637214000
O	0.107900000	2.962306000	-1.589491000
O	0.969085000	-0.982494000	0.055462000
O	-2.629315000	1.133572000	1.270276000
N	2.591853000	2.257570000	1.275527000
N	5.566695000	1.472394000	-0.764841000
N	0.615223000	1.267950000	3.375140000
N	0.000453000	3.647433000	2.384768000
C	3.380295000	1.778827000	0.294447000
H	2.879740000	1.237608000	-0.505396000
C	4.770988000	1.968148000	0.229216000
C	5.378727000	2.772875000	1.258871000
C	4.536926000	3.284499000	2.263583000
H	4.947453000	3.894731000	3.067800000
C	3.188179000	2.994609000	2.252936000
H	2.540653000	3.368780000	3.042218000
C	5.209743000	0.433216000	-1.528299000
C	5.978602000	0.058712000	-2.664348000
C	7.126424000	0.844325000	-3.083215000
C	7.919027000	0.462896000	-4.202709000
C	7.548083000	-0.676770000	-4.894190000
H	8.139471000	-0.984255000	-5.758591000

C	6.424849000	-1.468126000	-4.520906000
C	5.661736000	-1.085262000	-3.417441000
H	4.802652000	-1.689769000	-3.120671000
C	0.973216000	0.036561000	3.820909000
H	1.225234000	-0.693108000	3.053434000
C	1.011239000	-0.296892000	5.157193000
H	1.297704000	-1.296347000	5.479584000
C	0.659140000	0.673414000	6.115248000
C	0.292728000	1.939022000	5.671251000
H	0.017944000	2.699382000	6.398384000
C	0.273871000	2.227482000	4.301878000
C	-0.089445000	3.528127000	3.754980000
C	-0.520897000	4.604588000	4.533758000
H	-0.626149000	4.519044000	5.614012000
C	-0.853563000	5.816854000	3.940992000
C	-0.721767000	5.939212000	2.544457000
H	-0.962964000	6.870605000	2.037854000
C	-0.300255000	4.841885000	1.818632000
H	-0.208238000	4.891652000	0.734862000
C	0.219001000	2.516144000	-0.522298000
C	0.748390000	0.069074000	0.503909000
C	-1.499997000	1.386431000	1.280532000
C	6.091277000	-2.692468000	-5.311934000
H	6.939426000	-3.394848000	-5.317611000
H	5.211638000	-3.212896000	-4.917261000
H	5.900322000	-2.433617000	-6.365246000
C	9.095388000	1.303788000	-4.590772000
H	8.781983000	2.327043000	-4.844259000

H	9.806374000	1.396201000	-3.756982000
H	9.617367000	0.873771000	-5.452520000
N	6.705818000	2.991186000	1.245921000
H	7.156696000	3.539928000	1.966854000
H	7.284819000	2.534953000	0.550808000
H	4.340028000	-0.184848000	-1.273454000
C	0.682551000	0.281587000	7.558883000
O	0.998652000	-0.832814000	7.931526000
O	0.315764000	1.302189000	8.363725000
C	-1.362605000	6.909732000	4.828506000
O	-1.480561000	6.783677000	6.033931000
O	-1.670703000	8.016202000	4.121757000
C	-2.255191000	9.126762000	4.877862000
H	-1.820712000	9.124718000	5.884429000
H	-1.927057000	10.018406000	4.331408000
C	0.250992000	1.001657000	9.796277000
H	1.040439000	0.277827000	10.031147000
H	0.471224000	1.962169000	10.276098000
C	-3.765552000	9.011010000	4.915895000
H	-4.185239000	8.975480000	3.902457000
H	-4.078889000	8.115699000	5.466985000
H	-4.183285000	9.888433000	5.428172000
C	-1.120307000	0.480787000	10.176094000
H	-1.905635000	1.196273000	9.900729000
H	-1.323918000	-0.482343000	9.692253000
H	-1.161777000	0.330063000	11.263520000

Complex R6

Ground state (S_0)

Re	0.230218000	-0.148325000	-0.069756000
O	6.812471000	-3.300736000	3.016574000
H	6.316247000	-2.700167000	2.378796000
O	0.166587000	-0.240892000	3.021131000
O	0.331176000	2.944859000	-0.000533000
O	-2.864771000	-0.022931000	-0.035525000
N	2.474716000	-0.251460000	-0.089240000
N	5.193955000	-1.445563000	2.122557000
N	0.220729000	-0.378491000	-2.249627000
N	0.298496000	-2.307881000	-0.429925000
C	3.147650000	-0.795316000	0.955298000
H	2.549325000	-1.246888000	1.744851000
C	4.532232000	-0.834975000	1.062073000
C	5.311927000	-0.297174000	-0.007779000
C	4.605091000	0.266510000	-1.086094000
H	5.140509000	0.711080000	-1.924945000
C	3.224092000	0.277015000	-1.085146000
H	2.683182000	0.727989000	-1.915213000
C	4.791527000	-1.286748000	3.356958000
C	5.359387000	-2.007553000	4.455159000
C	6.358068000	-3.004040000	4.246303000
C	6.875300000	-3.693225000	5.351046000
C	6.420862000	-3.397782000	6.626166000
H	6.840375000	-3.946054000	7.472337000
C	5.435095000	-2.414134000	6.871283000

C	4.922076000	-1.739455000	5.775862000
H	4.156806000	-0.972800000	5.920238000
C	0.096087000	0.646859000	-3.122183000
H	0.082618000	1.645486000	-2.688388000
C	-0.028630000	0.462350000	-4.490890000
H	-0.138972000	1.320758000	-5.150057000
C	-0.023315000	-0.840142000	-4.997614000
C	0.115707000	-1.900276000	-4.105279000
H	0.107218000	-2.911156000	-4.508860000
C	0.233888000	-1.653942000	-2.738092000
C	0.324048000	-2.721620000	-1.730092000
C	0.399993000	-4.077232000	-2.057261000
H	0.421612000	-4.399955000	-3.095199000
C	0.446895000	-5.032867000	-1.044493000
C	0.413900000	-4.599588000	0.283110000
H	0.443301000	-5.328341000	1.092030000
C	0.339018000	-3.241852000	0.545857000
H	0.296176000	-2.869955000	1.568011000
C	0.196540000	-0.185205000	1.859983000
C	0.289096000	1.785142000	-0.003002000
C	-1.707387000	-0.074157000	-0.050786000
C	4.979185000	-2.125419000	8.275777000
H	5.810834000	-1.756484000	8.893754000
H	4.185472000	-1.368751000	8.293032000
H	4.594305000	-3.032809000	8.763078000
N	6.657982000	-0.329149000	0.059764000
H	7.235251000	0.044706000	-0.681074000
H	7.106974000	-0.731285000	0.874352000

H	3.989596000	-0.566316000	3.590968000
H	7.636026000	-4.455187000	5.182417000
C	-0.185868000	-1.171752000	-6.457220000
O	-0.190837000	-2.316174000	-6.867043000
O	-0.319092000	-0.061272000	-7.199373000
C	0.525672000	-6.513286000	-1.307933000
O	0.605437000	-7.328217000	-0.411381000
O	0.487439000	-6.782653000	-2.624582000
C	0.556191000	-8.201103000	-3.002075000
H	0.069546000	-8.786410000	-2.213298000
H	-0.034537000	-8.252310000	-3.923561000
C	-0.521513000	-0.255675000	-8.642093000
H	-0.038901000	0.619338000	-9.091730000
H	0.006631000	-1.168838000	-8.940248000
C	1.990715000	-8.632144000	-3.222652000
H	2.478606000	-8.012711000	-3.986030000
H	2.567926000	-8.581525000	-2.291251000
H	2.001722000	-9.673686000	-3.571769000
C	-1.998328000	-0.321261000	-8.970374000
H	-2.467615000	-1.201965000	-8.514876000
H	-2.520921000	0.582455000	-8.632162000
H	-2.120101000	-0.397492000	-10.059433000

Complex R6

Triplet state (T_1)

Re	0.187956000	-0.094619000	-0.068579000
O	7.167992000	-2.567933000	2.960961000
H	6.524113000	-1.999114000	2.390026000

O	0.148838000	-0.161792000	3.023220000
O	0.262454000	3.008390000	-0.035687000
O	-2.908334000	0.025631000	-0.041090000
N	2.433022000	-0.163917000	-0.064684000
N	5.196921000	-1.100863000	2.191865000
N	0.212798000	-0.344605000	-2.242693000
N	0.260400000	-2.247962000	-0.385625000
C	3.108483000	-0.651612000	0.988307000
H	2.518728000	-1.130384000	1.766799000
C	4.511784000	-0.604173000	1.127324000
C	5.262585000	-0.021618000	0.040749000
C	4.539202000	0.450971000	-1.069733000
H	5.059587000	0.898591000	-1.916249000
C	3.162013000	0.382120000	-1.080003000
H	2.596317000	0.774250000	-1.922054000
C	4.626642000	-1.315392000	3.389745000
C	5.304561000	-2.036168000	4.405074000
C	6.601649000	-2.649725000	4.160326000
C	7.265874000	-3.344679000	5.188726000
C	6.677307000	-3.463150000	6.429417000
H	7.191002000	-4.009399000	7.221232000
C	5.401845000	-2.880435000	6.705816000
C	4.748281000	-2.185261000	5.694094000
H	3.777227000	-1.729582000	5.897478000
C	0.102647000	0.663426000	-3.142083000
H	0.061986000	1.669685000	-2.726983000
C	0.026088000	0.458106000	-4.506657000
H	-0.073995000	1.303741000	-5.182808000

C	0.062109000	-0.861883000	-4.993372000
C	0.180505000	-1.901429000	-4.079460000
H	0.194183000	-2.919117000	-4.465641000
C	0.250755000	-1.638426000	-2.708429000
C	0.304578000	-2.680879000	-1.690548000
C	0.362330000	-4.047537000	-1.989490000
H	0.398282000	-4.384903000	-3.022422000
C	0.370052000	-4.988800000	-0.967241000
C	0.309634000	-4.533750000	0.363175000
H	0.302490000	-5.251265000	1.181624000
C	0.253978000	-3.177090000	0.602889000
H	0.190022000	-2.790538000	1.618759000
C	0.160331000	-0.115541000	1.859384000
C	0.233683000	1.848171000	-0.029400000
C	-1.752046000	-0.023951000	-0.056869000
C	4.808021000	-3.024023000	8.070178000
H	5.481796000	-2.604992000	8.833843000
H	3.836292000	-2.524725000	8.152590000
H	4.677468000	-4.087239000	8.326284000
N	6.600451000	0.070523000	0.127864000
H	7.148540000	0.500264000	-0.606758000
H	7.067661000	-0.205359000	0.984209000
H	3.640626000	-0.897684000	3.627517000
H	8.237050000	-3.787230000	4.968309000
C	-0.050436000	-1.218978000	-6.444231000
O	-0.032837000	-2.368083000	-6.845544000
O	-0.171241000	-0.119355000	-7.214061000
C	0.434484000	-6.465139000	-1.209670000

O	0.507114000	-7.275479000	-0.305075000
O	0.394732000	-6.759016000	-2.525939000
C	0.450923000	-8.180512000	-2.875779000
H	-0.033484000	-8.749987000	-2.073927000
H	-0.144995000	-8.248295000	-3.793160000
C	-0.335300000	-0.345340000	-8.652898000
H	0.148147000	0.524627000	-9.112103000
H	0.207811000	-1.259103000	-8.921644000
C	1.881227000	-8.627598000	-3.097678000
H	2.368872000	-8.024299000	-3.874178000
H	2.463706000	-8.562520000	-2.170311000
H	1.884568000	-9.675368000	-3.427996000
C	-1.803227000	-0.434281000	-9.017243000
H	-2.272902000	-1.310764000	-8.553961000
H	-2.343478000	0.469597000	-8.708048000
H	-1.899485000	-0.533540000	-10.107069000

Complex R7

Ground state (S_0)

Re	-0.416278000	0.211436000	2.128369000
O	3.187809000	3.091140000	-4.674202000
H	3.857397000	3.207515000	-5.385617000
O	2.665720000	0.421517000	2.317485000
O	-0.703726000	3.290798000	2.104510000
O	-0.546827000	0.294105000	5.222197000
N	-0.293015000	0.177175000	-0.115173000
N	1.316785000	2.168757000	-2.807593000
N	-2.553038000	-0.254707000	1.987367000

N	-0.550083000	-1.979332000	2.160608000
C	0.403984000	1.150516000	-0.760235000
H	0.897178000	1.890572000	-0.132588000
C	0.559290000	1.216603000	-2.138656000
C	-0.004613000	0.171252000	-2.937957000
C	-0.739823000	-0.821155000	-2.264321000
H	-1.210592000	-1.634371000	-2.816499000
C	-0.862031000	-0.777198000	-0.888399000
H	-1.429353000	-1.550277000	-0.372731000
C	1.334139000	3.397063000	-2.416858000
C	2.143529000	4.458323000	-2.992637000
C	3.039612000	4.292045000	-4.080494000
C	3.763770000	5.415686000	-4.524214000
C	3.628506000	6.667408000	-3.940249000
H	4.205645000	7.512629000	-4.311289000
C	2.739749000	6.820469000	-2.872132000
C	2.010535000	5.735972000	-2.409544000
H	1.321088000	5.867144000	-1.575607000
C	-3.524340000	0.677370000	1.867478000
H	-3.189846000	1.704719000	1.736812000
C	-4.872231000	0.366098000	1.927766000
H	-5.632657000	1.140934000	1.839997000
C	-5.254810000	-0.963341000	2.123057000
C	-4.260256000	-1.934555000	2.220971000
H	-4.543368000	-2.974053000	2.366694000
C	-2.916431000	-1.561093000	2.144754000
C	-1.803368000	-2.519237000	2.242090000
C	-1.985071000	-3.890278000	2.415491000

H	-2.975739000	-4.334473000	2.493360000
C	-0.881592000	-4.735247000	2.507676000
C	0.395614000	-4.178218000	2.401834000
H	1.285879000	-4.800686000	2.462368000
C	0.515535000	-2.806808000	2.228875000
H	1.493723000	-2.335279000	2.154752000
C	1.508042000	0.365881000	2.245916000
C	-0.571739000	2.135669000	2.112483000
C	-0.504604000	0.259987000	4.064565000
N	0.176745000	0.207413000	-4.272120000
H	-0.106157000	-0.561387000	-4.864844000
H	0.818905000	0.908887000	-4.634076000
H	0.686205000	3.734624000	-1.585946000
C	-6.725133000	-1.269224000	2.230343000
O	-7.575075000	-0.407709000	2.131091000
O	-6.945373000	-2.578748000	2.443204000
C	-1.143799000	-6.201799000	2.725394000
O	-2.268959000	-6.658167000	2.788509000
O	0.000507000	-6.894928000	2.832936000
C	-0.130846000	-8.340060000	3.068093000
H	-1.034292000	-8.687058000	2.553073000
H	0.758997000	-8.761280000	2.587166000
C	-8.351254000	-2.981543000	2.592474000
H	-8.955903000	-2.356565000	1.924671000
H	-8.360177000	-4.019721000	2.242119000
C	-0.172575000	-8.643548000	4.551008000
H	0.718435000	-8.256192000	5.061064000
H	-1.069866000	-8.219906000	5.018375000

H	-0.199103000	-9.732630000	4.692154000
C	-8.798942000	-2.863565000	4.034251000
H	-8.162148000	-3.462066000	4.698014000
H	-8.790145000	-1.818905000	4.368216000
H	-9.828010000	-3.237996000	4.121058000
Cl	2.557322000	8.382250000	-2.128775000
Cl	4.861242000	5.185030000	-5.862246000

Complex R7

Triplet state (T_1)

Re	-0.347816000	0.174790000	2.085581000
O	3.079888000	3.060023000	-4.493534000
H	3.742579000	3.125607000	-5.221009000
O	2.740361000	0.411278000	2.213840000
O	-0.591010000	3.258968000	2.072622000
O	-0.430162000	0.221553000	5.182018000
N	-0.259454000	0.172696000	-0.161122000
N	0.923155000	2.323619000	-2.902912000
N	-2.479932000	-0.276052000	1.938570000
N	-0.487299000	-2.013309000	2.120125000
C	0.329442000	1.187898000	-0.809770000
H	0.785067000	1.965535000	-0.199893000
C	0.393545000	1.300227000	-2.222529000
C	-0.132235000	0.187539000	-2.996596000
C	-0.742844000	-0.867178000	-2.295162000
H	-1.171880000	-1.714696000	-2.829202000
C	-0.802588000	-0.831512000	-0.916646000
H	-1.283373000	-1.638164000	-0.368378000

C	1.140777000	3.532649000	-2.375544000
C	2.023655000	4.515030000	-2.892822000
C	2.954579000	4.290247000	-3.968609000
C	3.732684000	5.363637000	-4.440946000
C	3.666139000	6.629418000	-3.880612000
H	4.284445000	7.440970000	-4.257958000
C	2.779734000	6.834988000	-2.801357000
C	1.987212000	5.813297000	-2.318005000
H	1.301944000	6.006194000	-1.493444000
C	-3.452999000	0.660800000	1.816884000
H	-3.113979000	1.687158000	1.688500000
C	-4.797885000	0.357899000	1.868194000
H	-5.553738000	1.135956000	1.775484000
C	-5.190531000	-0.977390000	2.062393000
C	-4.201219000	-1.949323000	2.172742000
H	-4.489474000	-2.987156000	2.321239000
C	-2.851410000	-1.588196000	2.105746000
C	-1.752401000	-2.544727000	2.212968000
C	-1.936217000	-3.914974000	2.408589000
H	-2.930030000	-4.350651000	2.495384000
C	-0.840867000	-4.764922000	2.515139000
C	0.447011000	-4.214880000	2.398955000
H	1.333081000	-4.841260000	2.471867000
C	0.573294000	-2.851300000	2.202964000
H	1.555056000	-2.387707000	2.122376000
C	1.581917000	0.338902000	2.168080000
C	-0.482738000	2.100215000	2.083173000
C	-0.407478000	0.198846000	4.024927000

N	-0.009273000	0.241359000	-4.326977000
H	-0.352081000	-0.493145000	-4.933410000
H	0.466862000	1.052502000	-4.719499000
H	0.532438000	3.856714000	-1.517920000
C	-6.655938000	-1.276383000	2.155567000
O	-7.507251000	-0.415050000	2.043669000
O	-6.888954000	-2.587170000	2.372711000
C	-1.110620000	-6.219520000	2.759942000
O	-2.235050000	-6.684045000	2.796205000
O	0.031697000	-6.911936000	2.930459000
C	-0.115111000	-8.343938000	3.212927000
H	-0.976524000	-8.717377000	2.646400000
H	0.808873000	-8.781866000	2.818919000
C	-8.296352000	-2.975285000	2.511503000
H	-8.891345000	-2.350890000	1.834269000
H	-8.313635000	-4.016560000	2.170005000
C	-0.269603000	-8.591804000	4.699358000
H	0.578560000	-8.178844000	5.260146000
H	-1.200974000	-8.154343000	5.078623000
H	-0.304026000	-9.674587000	4.881555000
C	-8.758115000	-2.842271000	3.948210000
H	-8.131457000	-3.439635000	4.622658000
H	-8.742425000	-1.794730000	4.272886000
H	-9.791051000	-3.207298000	4.029703000
Cl	2.688103000	8.414161000	-2.095077000
Cl	4.825944000	5.033086000	-5.759456000

Complex R8

Ground state (S_0)

Re	0.346419000	-0.203808000	2.297524000
O	2.934870000	2.852058000	-4.614179000
H	2.364737000	2.251979000	-4.039483000
O	3.429327000	-0.421687000	2.109653000
O	0.495651000	2.882426000	2.409245000
O	0.564102000	-0.224937000	5.386991000
N	0.179503000	-0.150199000	0.053190000
N	1.343341000	2.049194000	-2.693539000
N	-1.836149000	-0.362575000	2.304483000
N	-0.088456000	-2.354665000	2.320645000
C	0.781769000	0.851927000	-0.635943000
H	1.404341000	1.532077000	-0.057870000
C	0.685332000	1.029668000	-2.010442000
C	-0.055534000	0.071045000	-2.769381000
C	-0.658772000	-0.976597000	-2.048668000
H	-1.239241000	-1.739647000	-2.567041000
C	-0.528918000	-1.043914000	-0.675252000
H	-1.004277000	-1.855783000	-0.127885000
C	1.469523000	3.241027000	-2.173355000
C	2.251170000	4.275152000	-2.785308000
C	2.967131000	4.043745000	-3.997722000
C	3.722350000	5.082463000	-4.559852000
C	3.779001000	6.320290000	-3.939569000
H	4.369508000	7.124701000	-4.377280000
C	3.080970000	6.552258000	-2.740636000

C	2.323716000	5.547094000	-2.171053000
H	1.782626000	5.727743000	-1.241686000
C	-2.671267000	0.696704000	2.223507000
H	-2.200727000	1.676321000	2.167843000
C	-4.049324000	0.563766000	2.217345000
H	-4.698515000	1.435602000	2.148908000
C	-4.609491000	-0.712940000	2.305279000
C	-3.755712000	-1.810898000	2.392504000
H	-4.177133000	-2.811045000	2.457714000
C	-2.372826000	-1.614658000	2.387556000
C	-1.399367000	-2.716785000	2.458895000
C	-1.758642000	-4.046602000	2.670384000
H	-2.796118000	-4.348002000	2.804668000
C	-0.776945000	-5.032839000	2.739156000
C	0.558635000	-4.657800000	2.571416000
H	1.357922000	-5.394812000	2.611782000
C	0.856968000	-3.318964000	2.364871000
H	1.886232000	-2.986130000	2.243584000
C	2.275589000	-0.318411000	2.184754000
C	0.464144000	1.720824000	2.362119000
C	0.477456000	-0.221002000	4.231665000
N	-0.165282000	0.202991000	-4.106535000
H	-0.649003000	-0.485045000	-4.667524000
H	0.294101000	0.975980000	-4.573593000
H	0.959337000	3.497306000	-1.230112000
C	-6.110416000	-0.828069000	2.298131000
O	-6.833552000	0.139578000	2.174783000
O	-6.511238000	-2.103469000	2.439076000

C	-1.224348000	-6.446182000	3.004874000
O	-2.396631000	-6.743500000	3.129534000
O	-0.179546000	-7.284540000	3.079767000
C	-0.487501000	-8.693017000	3.368534000
H	-1.451385000	-8.931983000	2.904231000
H	0.316603000	-9.240661000	2.864670000
C	-7.965085000	-2.319912000	2.481893000
H	-8.432953000	-1.613974000	1.785576000
H	-8.083256000	-3.344307000	2.111767000
C	-0.498805000	-8.947037000	4.861217000
H	0.458476000	-8.667512000	5.319089000
H	-1.309534000	-8.394953000	5.352162000
H	-0.661580000	-10.018510000	5.040323000
C	-8.496665000	-2.158401000	3.890686000
H	-7.993982000	-2.843053000	4.585551000
H	-8.374535000	-1.127627000	4.244831000
H	-9.569572000	-2.393544000	3.897457000
Cl	3.182019000	8.115194000	-1.978848000
H	4.262699000	4.894600000	-5.487265000

Complex R8

Triplet state (T_1)

Re	0.440323000	-0.273767000	2.351620000
O	2.486086000	2.997322000	-4.665248000
H	1.977516000	2.396543000	-3.989120000
O	3.532426000	-0.510794000	2.271374000
O	0.672798000	2.806428000	2.497562000
O	0.538209000	-0.327873000	5.448082000

N	0.347262000	-0.199207000	0.105389000
N	1.192896000	2.134821000	-2.628379000
N	-1.737124000	-0.389034000	2.276557000
N	-0.033822000	-2.415674000	2.352960000
C	0.798453000	0.880346000	-0.559498000
H	1.267886000	1.655968000	0.040095000
C	0.737592000	1.040703000	-1.958598000
C	0.184166000	-0.050969000	-2.724115000
C	-0.255476000	-1.183190000	-2.014641000
H	-0.677076000	-2.036137000	-2.546214000
C	-0.177991000	-1.213930000	-0.637342000
H	-0.534807000	-2.081347000	-0.087706000
C	1.421077000	3.316972000	-2.030819000
C	2.071948000	4.369233000	-2.725021000
C	2.588695000	4.180439000	-4.074310000
C	3.208691000	5.241127000	-4.763088000
C	3.355364000	6.472819000	-4.159163000
H	3.839586000	7.299714000	-4.675343000
C	2.864904000	6.661205000	-2.838819000
C	2.239128000	5.639146000	-2.136678000
H	1.870173000	5.822399000	-1.127791000
C	-2.554623000	0.687372000	2.170270000
H	-2.063058000	1.656455000	2.108428000
C	-3.930154000	0.585436000	2.148232000
H	-4.559141000	1.469608000	2.060075000
C	-4.522870000	-0.684350000	2.252114000
C	-3.693262000	-1.795754000	2.367012000
H	-4.136391000	-2.785547000	2.446056000

C	-2.304018000	-1.636052000	2.374862000
C	-1.363462000	-2.749600000	2.467607000
C	-1.749665000	-4.075653000	2.669003000
H	-2.796194000	-4.353626000	2.782595000
C	-0.794520000	-5.083642000	2.752755000
C	0.559984000	-4.734832000	2.613089000
H	1.341848000	-5.488800000	2.668209000
C	0.889010000	-3.405952000	2.419160000
H	1.928322000	-3.095929000	2.323246000
C	2.376798000	-0.406271000	2.305502000
C	0.605654000	1.644985000	2.437847000
C	0.495456000	-0.313875000	4.291508000
N	0.089997000	0.045627000	-4.061709000
H	-0.302150000	-0.703760000	-4.617774000
H	0.344220000	0.910032000	-4.525394000
H	1.073062000	3.515549000	-1.010490000
C	-6.018255000	-0.767325000	2.235038000
O	-6.726080000	0.210876000	2.087662000
O	-6.454000000	-2.033286000	2.398917000
C	-1.274758000	-6.481349000	3.004056000
O	-2.453867000	-6.764460000	3.110965000
O	-0.246545000	-7.346813000	3.092001000
C	-0.592612000	-8.742645000	3.379355000
H	-1.555231000	-8.961935000	2.902482000
H	0.204712000	-9.314468000	2.891126000
C	-7.909178000	-2.209944000	2.441303000
H	-8.359807000	-1.504747000	1.732652000
H	-8.056148000	-3.237351000	2.089346000

C	-0.633871000	-8.992664000	4.873125000
H	0.321852000	-8.731668000	5.345256000
H	-1.439215000	-8.419218000	5.348283000
H	-0.823733000	-10.059312000	5.054752000
C	-8.441227000	-2.010607000	3.845858000
H	-7.957836000	-2.696267000	4.553443000
H	-8.290212000	-0.977780000	4.182571000
H	-9.520281000	-2.215923000	3.854911000
Cl	3.050033000	8.209464000	-2.104952000
H	3.575812000	5.056455000	-5.772288000

Complex R9

Ground state (S_0)

Re	0.316348000	-1.041172000	-1.840783000
O	7.693581000	-4.535558000	-0.534052000
H	8.469575000	-5.120627000	-0.643381000
O	0.302350000	-3.858823000	-3.124378000
O	0.558292000	-2.248983000	0.995152000
O	-2.761639000	-1.200591000	-1.529982000
N	2.554759000	-0.933867000	-2.055610000
N	5.548388000	-2.726599000	-0.779855000
N	0.403338000	1.061507000	-1.239104000
N	0.058400000	0.113282000	-3.691538000
C	3.344461000	-1.801113000	-1.368344000
H	2.834693000	-2.547961000	-0.762724000
C	4.731698000	-1.816808000	-1.437455000
C	5.380424000	-0.905587000	-2.330317000
C	4.557152000	-0.003719000	-3.028383000

H	4.988484000	0.727369000	-3.711950000
C	3.186536000	-0.045655000	-2.859241000
H	2.556342000	0.651932000	-3.407176000
C	5.265115000	-3.129509000	0.411424000
C	6.005719000	-4.130203000	1.158523000
C	7.160892000	-4.797138000	0.684372000
C	7.758743000	-5.749745000	1.520797000
C	7.289746000	-6.071781000	2.779278000
H	7.788776000	-6.819901000	3.393162000
C	6.153742000	-5.396227000	3.224706000
C	5.519349000	-4.447819000	2.447822000
H	4.632276000	-3.945498000	2.832748000
C	0.636899000	1.477951000	0.024941000
H	0.790001000	0.696829000	0.767068000
C	0.673592000	2.816388000	0.377248000
H	0.863436000	3.122226000	1.405169000
C	0.455450000	3.784249000	-0.606311000
C	0.216035000	3.363853000	-1.912762000
H	0.048036000	4.105990000	-2.689262000
C	0.196508000	1.999129000	-2.208768000
C	-0.044782000	1.470409000	-3.560466000
C	-0.376237000	2.274266000	-4.649573000
H	-0.482548000	3.353719000	-4.557035000
C	-0.601129000	1.700575000	-5.898869000
C	-0.472685000	0.315084000	-6.029517000
H	-0.635558000	-0.175851000	-6.986636000
C	-0.143166000	-0.435131000	-4.909997000
H	-0.045443000	-1.517654000	-4.969677000

C	0.305994000	-2.812911000	-2.621078000
C	0.474095000	-1.816972000	-0.081724000
C	-1.610340000	-1.135332000	-1.646504000
N	6.722877000	-0.943141000	-2.432928000
H	7.213844000	-0.401379000	-3.131420000
H	7.202235000	-1.710438000	-1.966781000
H	4.406428000	-2.704189000	0.964273000
F	5.669504000	-5.686953000	4.452522000
F	8.864664000	-6.371922000	1.028391000
C	0.486464000	5.231865000	-0.195570000
O	0.737031000	5.577406000	0.941245000
O	0.206090000	6.046213000	-1.228257000
C	-0.983829000	2.619695000	-7.028569000
O	-1.094946000	3.821200000	-6.878773000
O	-1.171023000	1.941218000	-8.170885000
C	-1.578600000	2.729142000	-9.343136000
H	-1.121849000	3.722233000	-9.261679000
H	-1.140311000	2.183360000	-10.186094000
C	0.164300000	7.484130000	-0.923904000
H	0.930088000	7.692936000	-0.167590000
H	0.437746000	7.957193000	-1.873517000
C	-3.087773000	2.804767000	-9.438804000
H	-3.533438000	1.802720000	-9.474959000
H	-3.511953000	3.359220000	-8.592705000
H	-3.362221000	3.333011000	-10.362018000
C	-1.216544000	7.897319000	-0.458913000
H	-1.978709000	7.649163000	-1.208456000
H	-1.474275000	7.417314000	0.492934000

H	-1.232040000	8.984780000	-0.304640000

Complex R9

Triplet state (T_1)

Re	0.372438000	-1.064048000	-1.891929000
O	7.477163000	-4.469927000	-0.530475000
H	8.252296000	-5.047076000	-0.690067000
O	0.343630000	-3.889602000	-3.168212000
O	0.606241000	-2.309923000	0.928488000
O	-2.707035000	-1.204706000	-1.599685000
N	2.618149000	-0.983804000	-2.104761000
N	5.635051000	-2.380320000	-0.533946000
N	0.483281000	1.029593000	-1.273976000
N	0.115477000	0.106095000	-3.730886000
C	3.401951000	-1.726340000	-1.311860000
H	2.909325000	-2.382359000	-0.597475000
C	4.823685000	-1.705743000	-1.349855000
C	5.445092000	-0.895895000	-2.386800000
C	4.601636000	-0.133853000	-3.212384000
H	5.015014000	0.501658000	-3.995083000
C	3.233446000	-0.189359000	-3.035524000
H	2.576933000	0.403467000	-3.667027000
C	5.245814000	-2.945992000	0.618442000
C	5.908495000	-4.015697000	1.266896000
C	7.022331000	-4.736619000	0.714023000
C	7.628806000	-5.739933000	1.480680000
C	7.195572000	-6.112951000	2.735187000
H	7.683585000	-6.905776000	3.298407000

C	6.084099000	-5.417071000	3.245921000
C	5.451606000	-4.410095000	2.556457000
H	4.604319000	-3.899464000	3.012017000
C	0.726889000	1.440126000	-0.005614000
H	0.895425000	0.653416000	0.727529000
C	0.752956000	2.770932000	0.360731000
H	0.950527000	3.067269000	1.389595000
C	0.507177000	3.751963000	-0.612817000
C	0.254132000	3.341290000	-1.918201000
H	0.063389000	4.089096000	-2.684103000
C	0.246661000	1.979874000	-2.235068000
C	-0.001874000	1.467505000	-3.581174000
C	-0.352970000	2.276108000	-4.662515000
H	-0.469691000	3.353294000	-4.555896000
C	-0.586716000	1.715950000	-5.914664000
C	-0.445491000	0.326326000	-6.062336000
H	-0.616723000	-0.155880000	-7.022035000
C	-0.097839000	-0.429551000	-4.957240000
H	0.005716000	-1.511002000	-5.029769000
C	0.351798000	-2.839744000	-2.672723000
C	0.522900000	-1.861214000	-0.143042000
C	-1.555871000	-1.144354000	-1.709132000
N	6.777727000	-0.928447000	-2.486084000
H	7.283008000	-0.391860000	-3.179938000
H	7.279600000	-1.534430000	-1.838152000
H	4.397499000	-2.511749000	1.166849000
F	5.643105000	-5.760811000	4.471297000
F	8.686799000	-6.371317000	0.904859000

C	0.520016000	5.189850000	-0.188535000
O	0.776007000	5.532757000	0.949608000
O	0.216046000	6.015906000	-1.209882000
C	-0.993305000	2.637847000	-7.025535000
O	-1.106353000	3.840300000	-6.874331000
O	-1.205339000	1.964218000	-8.171431000
C	-1.645794000	2.758956000	-9.323110000
H	-1.185194000	3.751168000	-9.250982000
H	-1.236281000	2.218547000	-10.184078000
C	0.149460000	7.444929000	-0.883978000
H	0.916628000	7.659379000	-0.130417000
H	0.405145000	7.939369000	-1.827887000
C	-3.157533000	2.841170000	-9.373964000
H	-3.607791000	1.840810000	-9.402053000
H	-3.553408000	3.391145000	-8.511387000
H	-3.458534000	3.376366000	-10.284868000
C	-1.235307000	7.825418000	-0.401192000
H	-1.998915000	7.570668000	-1.147133000
H	-1.474252000	7.326800000	0.546034000
H	-1.272367000	8.910234000	-0.232080000

Complex R10

Ground state (S_0)

Re	0.102195000	0.304870000	-0.069822000
O	7.201350000	2.760211000	-2.906680000
H	6.573441000	2.253381000	-2.301949000
O	0.136648000	0.621766000	-3.146356000
O	0.050244000	-2.778425000	-0.350601000

O	-2.992628000	0.335114000	-0.188053000
N	2.350727000	0.261529000	0.014000000
N	5.218953000	1.261320000	-2.108441000
N	0.062653000	0.391520000	2.117959000
N	0.248487000	2.429172000	0.436273000
C	3.092475000	0.787042000	-0.991306000
H	2.554286000	1.335629000	-1.762552000
C	4.475865000	0.687765000	-1.080403000
C	5.180148000	0.024032000	-0.029175000
C	4.401649000	-0.510876000	1.014314000
H	4.875204000	-1.044022000	1.838624000
C	3.027640000	-0.384291000	0.993537000
H	2.430980000	-0.819089000	1.793023000
C	4.795861000	1.252154000	-3.344131000
C	5.495522000	1.922098000	-4.401423000
C	6.691145000	2.659146000	-4.145820000
C	7.354860000	3.293090000	-5.206287000
C	6.851238000	3.208639000	-6.495160000
H	7.356372000	3.696102000	-7.328933000
C	5.675804000	2.487149000	-6.736414000
C	4.997859000	1.846989000	-5.723988000
H	4.087476000	1.288181000	-5.943122000
C	-0.106151000	-0.689206000	2.914077000
H	-0.169068000	-1.651621000	2.408923000
C	-0.211584000	-0.596736000	4.290906000
H	-0.356836000	-1.484082000	4.905637000
C	-0.140746000	0.661895000	4.893978000
C	0.035190000	1.780577000	4.082013000

H	0.080500000	2.766950000	4.537028000
C	0.131660000	1.625543000	2.696421000
C	0.260239000	2.755606000	1.762700000
C	0.350039000	4.085036000	2.173467000
H	0.359628000	4.362395000	3.226122000
C	0.419642000	5.106455000	1.228775000
C	0.402320000	4.763933000	-0.125589000
H	0.446742000	5.528342000	-0.898452000
C	0.318229000	3.424212000	-0.474270000
H	0.288132000	3.120006000	-1.519136000
C	0.126740000	0.477628000	-1.991935000
C	0.065070000	-1.621291000	-0.268711000
C	-1.835263000	0.330027000	-0.140890000
N	6.522985000	-0.082281000	-0.073608000
H	7.038754000	-0.562792000	0.651272000
H	7.030686000	0.283120000	-0.870354000
H	3.879842000	0.703540000	-3.619442000
H	8.268003000	3.848252000	-4.994503000
F	5.202528000	2.422561000	-8.003660000
C	-0.281315000	0.732977000	6.391502000
O	-0.436006000	-0.259534000	7.073812000
O	-0.224520000	1.999644000	6.837746000
C	0.492175000	6.524417000	1.731482000
O	0.498612000	6.794404000	2.916616000
O	0.548026000	7.401304000	0.716592000
C	0.594097000	8.824285000	1.082141000
H	1.149275000	8.915381000	2.023009000
H	1.163793000	9.281885000	0.265739000

C	-0.369730000	2.176306000	8.290489000
H	-0.777842000	3.188534000	8.385049000
H	-1.102766000	1.443268000	8.647931000
C	0.961515000	2.030596000	8.997026000
H	1.352044000	1.010528000	8.900112000
H	1.699181000	2.742316000	8.604956000
H	0.822177000	2.239574000	10.066435000
C	-0.803119000	9.397456000	1.191252000
H	-1.361758000	9.261556000	0.256605000
H	-1.359187000	8.934466000	2.015671000
H	-0.733316000	10.475053000	1.392508000

Complex R10

Triplet state (T_1)

Re	0.408879000	1.580868000	-0.513618000
O	7.704487000	1.059224000	-3.746559000
H	6.967670000	1.145946000	-3.018799000
O	0.437689000	2.572989000	-3.449866000
O	0.711632000	-1.333299000	-1.512283000
O	-2.671639000	1.282114000	-0.684116000
N	2.641715000	1.753427000	-0.386242000
N	5.617456000	0.715117000	-2.308645000
N	0.419999000	1.163756000	1.629521000
N	0.194093000	3.530964000	0.455824000
C	3.418210000	1.226141000	-1.350558000
H	2.899424000	0.794134000	-2.203282000
C	4.827952000	1.237361000	-1.332811000
C	5.468540000	1.887508000	-0.213300000

C	4.640150000	2.456814000	0.769848000
H	5.075546000	2.960504000	1.632700000
C	3.268044000	2.355475000	0.663977000
H	2.628912000	2.773542000	1.437423000
C	5.174747000	-0.167648000	-3.221573000
C	5.981585000	-0.527334000	-4.330442000
C	7.277920000	0.102585000	-4.561416000
C	8.087904000	-0.279899000	-5.650201000
C	7.651745000	-1.244044000	-6.535844000
H	8.246906000	-1.556705000	-7.392190000
C	6.387229000	-1.837384000	-6.314962000
C	5.564637000	-1.508147000	-5.253101000
H	4.606421000	-2.014149000	-5.138024000
C	0.569547000	-0.070648000	2.170041000
H	0.741069000	-0.882522000	1.465625000
C	0.506120000	-0.308680000	3.527505000
H	0.628568000	-1.313307000	3.928598000
C	0.274441000	0.768750000	4.400027000
C	0.113507000	2.039099000	3.856182000
H	-0.068880000	2.882106000	4.518204000
C	0.184521000	2.224369000	2.471311000
C	0.033352000	3.524333000	1.821306000
C	-0.271805000	4.702430000	2.506534000
H	-0.427250000	4.712030000	3.583967000
C	-0.408549000	5.902407000	1.817759000
C	-0.210003000	5.903493000	0.426927000
H	-0.299292000	6.819606000	-0.152253000
C	0.084509000	4.707956000	-0.203311000

H	0.225967000	4.665576000	-1.281937000
C	0.427520000	2.187970000	-2.353247000
C	0.588750000	-0.235098000	-1.149819000
C	-1.522134000	1.398070000	-0.613843000
N	6.809714000	1.913668000	-0.132527000
H	7.282399000	2.352502000	0.647757000
H	7.366716000	1.416375000	-0.817834000
H	4.204055000	-0.663962000	-3.110041000
H	9.050139000	0.215231000	-5.775360000
F	5.977080000	-2.772881000	-7.184328000
C	0.210771000	0.487390000	5.870540000
O	0.350418000	-0.629748000	6.330203000
O	-0.017898000	1.607858000	6.586098000
C	-0.782455000	7.121575000	2.608147000
O	-0.939868000	7.101978000	3.814725000
O	-0.916574000	8.200461000	1.814069000
C	-1.326852000	9.445716000	2.471226000
H	-0.907213000	9.453073000	3.483982000
H	-0.855179000	10.228118000	1.865737000
C	-0.095367000	1.438248000	8.040424000
H	-0.754638000	2.252412000	8.361857000
H	-0.569719000	0.471378000	8.246770000
C	1.276623000	1.536311000	8.675464000
H	1.921875000	0.711264000	8.350285000
H	1.758463000	2.491980000	8.431809000
H	1.172772000	1.476054000	9.767464000
C	-2.835971000	9.573569000	2.484548000
H	-3.246401000	9.525065000	1.467919000

H	-3.293998000	8.786706000	3.096433000
H	-3.111629000	10.544542000	2.918152000
